# Chiral Fluorescent Antifungal Azole Probes Detect Resistance, Uptake Dynamics, and Subcellular Distribution in *Candida* Species

**DOI:** 10.1021/jacsau.4c00479

**Published:** 2024-08-13

**Authors:** Vlad Koren, Efrat Ben-Zeev, Ivan Voronov, Micha Fridman

**Affiliations:** † School of Chemistry, Raymond and Beverley Sackler Faculty of Exact Sciences, Tel Aviv University, Tel Aviv 6997801, Israel; ‡ Ilana and Pascal Mantoux Institute for Bioinformatics and Nancy and Stephen Grand Israel National Center for Personalized Medicine, Weizmann Institute of Science, Rehovot 7610001, Israel

**Keywords:** fluorescent antifungal azoles, *Candida*, subcellular distribution, azole resistance, live-cell imaging, fluorescent probes

## Abstract

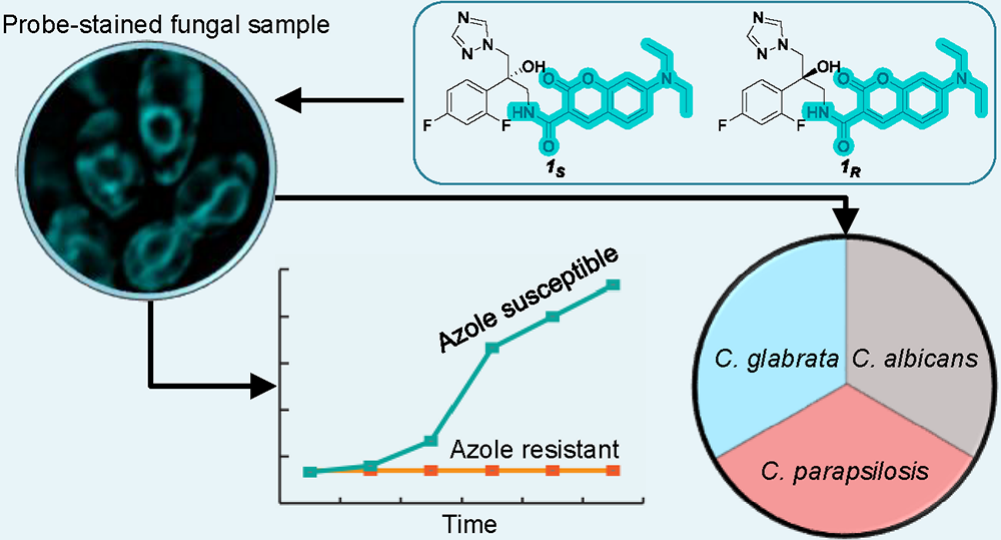

Azoles are essential for fungal infection treatment, yet the increasing resistance highlights the need for innovative diagnostic tools and strategies to revitalize this class of antifungals. We developed two enantiomers of a fluorescent antifungal azole probe (**1**_*S*_ and **1**_*R*_), analyzing 60 *Candida* strains via live-cell microscopy. A database of azole distribution images in strains of *Candida albicans*, *Candida glabrata*, and *Candida parapsilosis*, among the most important pathogenic *Candida* species, was established and analyzed. This analysis revealed distinct populations of yeast cells based on the correlation between fluorescent probe uptake and cell diameter. Varied uptake levels and subcellular distribution patterns were observed in *C. albicans*, *C. glabrata*, and *C. parapsilosis*, with the latter displaying increased localization to lipid droplets. Comparison of the more potent fluorescent antifungal azole probe enantiomer **1**_*S*_ with the moderately potent enantiomer **1**_*R*_ highlighted time-dependent differences in the uptake profiles. The former displayed a marked elevation in uptake after approximately 150 min, indicating the time required for significant cell permeabilization to occur and its association with the azole’s antifungal activity potency. Divergent uptake levels between susceptible and high efflux-based azole-resistant strains were detected, offering a rapid diagnostic approach for identifying azole resistance. This study highlights unique insights achievable through fluorescent antifungal azole probes, unraveling the complexities of azole resistance, subcellular dynamics, and uptake within fungal pathogens.

## Introduction

Antifungal azole drugs, encompassing imidazoles, triazoles, and the emerging tetrazoles, constitute the largest among the three primary classes of antifungal medications.^1−7^ This class is pivotal in the treatment and prevention of *Candida* infections, which are the most common type of fungal infections in humans and represent a major genus of medically important yeast.^8−10^ Additionally, it holds significant importance in global crop protection, constituting over one-third of all applied agricultural antifungal agents.^11,12^

Azoles exert their antifungal activity by inhibiting the biosynthesis of ergosterol, the principal sterol in fungal cell membranes.^13−15^ Specifically, they target the cytochrome P450 lanosterol 14α-demethylase (also termed Erg11 or Cyp51), impeding the oxidative removal of the 14α-methyl group from lanosterol, one of the intermediate sterols of ergosterol.^16^ The binding of azole rings to the heme cofactor at the catalytic site of Cyp51 inhibits the enzyme’s activity.^17^

Over recent decades, azole resistance has surged in *Candida* species.^18−21^ Resistance jeopardizes the management of fungal infections and food production globally.^22^ Mechanisms of resistance include mutations in the target Cyp51 gene and/or its overexpression and, mainly, increased efflux activity by ATP-binding cassette (ABC) and major facilitator superfamily (MFS) transport pumps.^23−25^*Candida glabrata*, one of the most prevalent pathogens in the genus *Candida*, exhibits intrinsically low susceptibility to currently used azole antifungals that is often the result of efflux pump overexpression.^26−28^ Resistance to azoles leads to increased treatment failure, especially in immunocompromised individuals.^29^

Despite extensive research, several aspects of azole potency remain unclear. One such aspect is the uptake and subcellular distribution of azole antifungals in fungal cells and their impact on the potency and resistance. A useful approach to exploring these aspects is to develop fluorescent probes for live-cell microscopy that maintain the mode of action and share molecular features similar to those of the parent compound. Fluorescent microscopy techniques are extensively utilized for studying biological processes within living cells, and they also serve as rapid, simplified diagnostic assays for infectious diseases in clinical microbiology laboratories worldwide.^30−34^ We previously developed and employed fluorescent antifungal azole probes to track azoles within live *Candida* yeast cells.^35^ Live-cell fluorescent imaging experiments have revealed that, depending on their structure, fluorescent azole probes exhibit a predominant localization to mitochondria, whereas the target enzyme Cyp51 was shown to be situated mainly in the endoplasmic reticulum (ER).^36,37^ We next designed and synthesized a fluorescent azole probe based on a 7-diethyl-aminocoumarin fluorophore, directing it mainly to the ER.^38,39^ In comparison to antifungal azoles that accumulate mainly in mitochondria, this fluorescent antifungal azole probe exhibited significantly higher antifungal potency *in vitro.*(40) This is indicative of the potential contribution of target-oriented subcellular localization in optimizing drug potency.

Here we report on our investigation of the intricate uptake and subcellular distribution of two enantiomers of an ER-localizing antifungal azole probe in three main *Candida* species. We delve into the interplay among probe uptake, time-dependent subcellular distribution, efficacy, and time needed for membrane damage and explore the interplay between these factors and azole resistance.

## Results and Discussion

### Design and Synthesis of Two Enantiomers of a 7-Diethylaminocoumarin-Based Fluorescent Antifungal Azole Probe and Evaluation of Their Antifungal Potency

**Figure 1:**
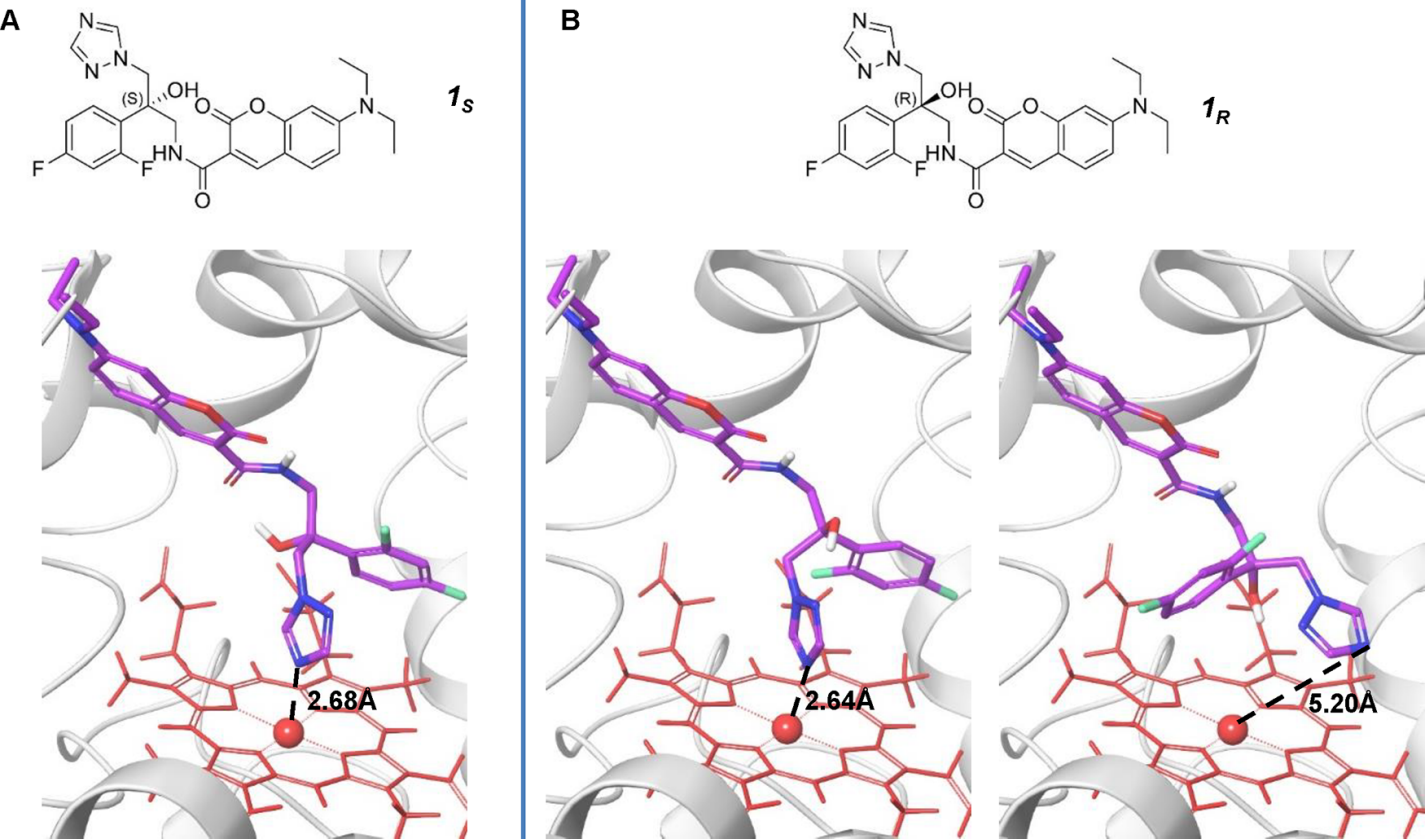
Chemical structure and docking of fluorescent azole probes **1**_*S*_ and **1**_*R*_ to *C. albicans* Cyp51, highlighting docking scores and triazole–heme distances.(A) The chemical structure and docking of fluorescent azole probe **1**_*S*_ yielded a score of −5.859 to *C. albicans* Cyp51 (PDB code 5FSA). (B) Docking of fluorescent azole probe **1**_*R*_ to *C. albicans* Cyp51. Notably, two different poses of azole probe **1**_*R*_ achieved high docking scores (−5.652 for the pose in the left image of panel B and a docking score of −4.889 for the pose in the right image of panel B). Distances between the basic triazole nitrogen and heme iron are presented in the background. Schrödinger Glide XP docking; parameters: saving 50 poses for the ligand. For the grid construction, the ligand (voriconazole) from structure 5FSA was used. Flexible MMGBSA was used to rerank the docking results.

In choosing the fluorescent antifungal probes for this study, we focused on the synthesis and evaluation of the *R* and *S* enantiomers of the fluorescent 7-diethylaminocoumarin-based antifungal azole (**1**_*S*_ and **1**_*R*_, respectively, Figure 1, Scheme S1). We prepared the two pure enantiomers **1**_*S*_ and **1**_*R*_ representing two antifungal azoles that differ structurally and chemically solely by their single chiral center and, as a result, in their antifungal potency.

In an *in silico* analysis of the interactions between the two enantiomers of the fluorescent azole probe (**1**_*S*_ and **1**_*R*_), docking computations were conducted to explore their binding with the target Cyp51. The results revealed that **1**_*R*_ can adapt to the catalytic domain of the enzyme in more than one pose. A single pose of **1**_*S*_ received the highest docking score, wherein its 1,2,4-triazole ring interacts with the heme iron of Cyp51 (Figure 1A), a characteristic shared with other antifungal triazoles previously cocrystallized with the target protein.^41^

Of note, two poses of **1**_*R*_ received similar high docking scores. Like the single pose of **1**_*S*_, in one pose of **1**_*R*_, the 1,2,4-triazole ring interacts with the heme iron of Cyp51 (left image, Figure 1B). However, in the other pose, the 1,2,4-triazole ring does not reside in proximity sufficient to interact with the heme iron (right image, Figure 1B). Moreover, in probe **1**_*S*_, the benzylic tertiary alcohol can form an intramolecular hydrogen bond with the amide carbonyl, which can stabilize this enantiomer in the pose that interacts with the heme iron atom of Cyp51 (Figure S1). This hydrogen bond is not formed in the two main poses of **1**_*R*_, making it a less rigid structure.

To conclude, docking computations suggest that although both enantiomers, **1**_*S*_ and **1**_*R*_, can effectively bind to the target Cyp51, the latter can adopt more than one pose in the catalytic pocket of the enzyme, one of which does not involve interaction with the heme iron. This lack of interaction likely contributes to the reduced efficacy of **1**_*R*_ in inhibiting target Cyp51.

The antifungal activity evaluation of the fluorescent antifungal azole enantiomers focused on the three main and most encountered fungal pathogens of the genus *Candida*. *C. albicans* is the most prevalent fungal species of the human microbiota;^42^ this species colonizes many areas of the body, particularly the gastrointestinal and genitourinary tracts of healthy individuals. *C. albicans* remains the most common opportunistic fungal pathogen isolated from fungal infections, yet its proportion relative to non-albicans *Candida* species has moderately decreased over time. In recent years, non-albicans *Candida* species have become more common, with the percentage of yeast infections caused by *C. glabrata* and *C. parapsilosis* growing rapidly.^43^ Depending on the geographic region, these species are identified as the second or third most prominent pathogens within the genus *Candida* following *C. albicans*.^44^

**Figure 2:**
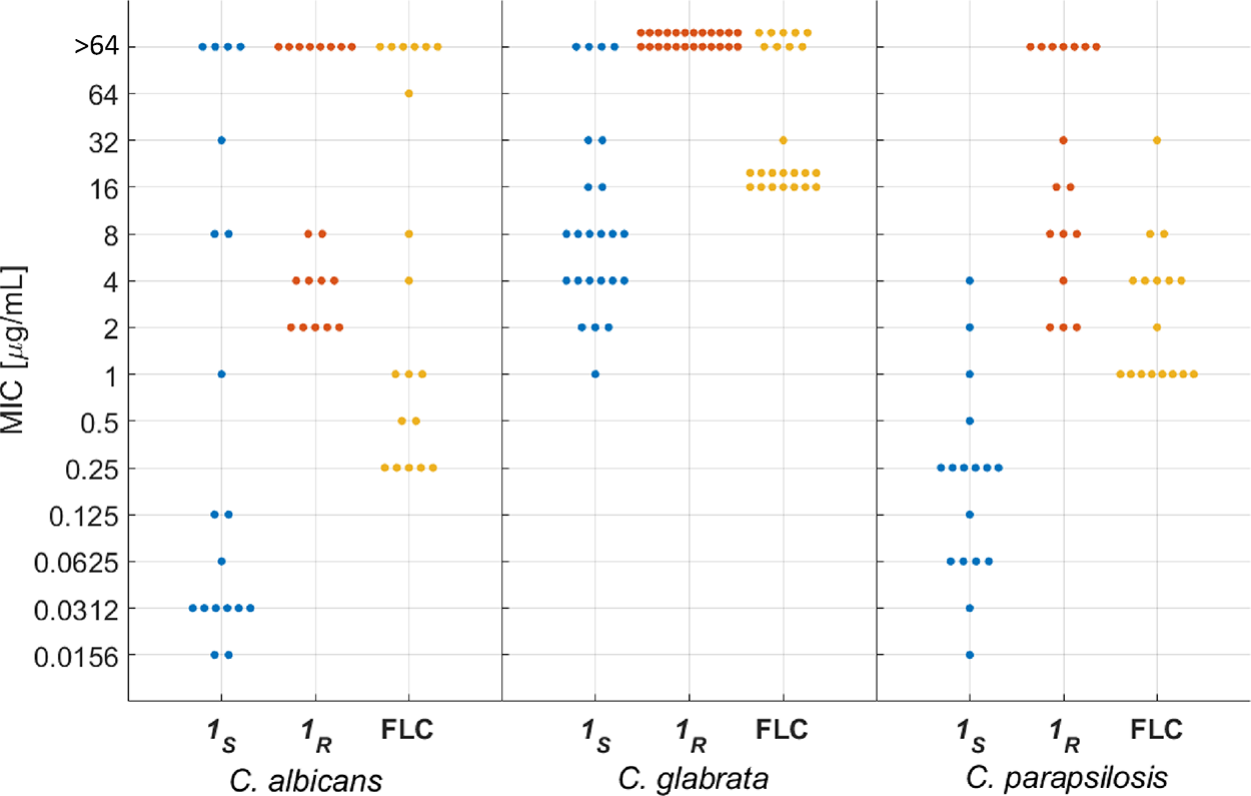
Antifungal activities of fluorescent azole probes **1**_*S*_ and **1**_*R*_ compared to FLC on a panel of 60 *Candida* strains. Minimal inhibitory concentration (MIC) values for **1**_*S*_ are denoted in blue, those for **1**_*R*_ are represented in orange, and those for FLC are indicated in yellow. Each colored dot represents a single fungal strain in the panel of 60 *Candida* strains in this study. Experiments were carried out in YPAD medium at 30 °C for 24 h. Each concentration was assessed in triplicate, and the results were validated in at least two independent experiments. MIC values for each antifungal azole against every strain in the panel are summarized in Tables S4–S6.

In this study, we assessed the antifungal activity of fluorescent antifungal azole probes **1**_*S*_ and **1**_*R*_ against a panel comprising 60 *Candida* strains, encompassing both azole-susceptible and azole-resistant strains, including 19 *C. albicans* strains (Table S1), 24 *C. glabrata* strains (Table S2), and 17 *C. parapsilosis* strains (Table S3). The results are summarized in Figure 2, and minimal inhibitory concentration (MIC) values, defined as the lowest concentration at which no visible growth was observed, for each strain are detailed in Tables S4–S6. As a control, we tested the clinically used antifungal azole drug fluconazole (FLC).

Among the 19 *C. albicans* strains, 11 showed susceptibility (MIC ≤ 0.5), whereas 8 exhibited varying levels of resistance (1 μg/mL ≤ MIC ≤ 64 μg/mL) to **1**_*S*_, the more potent enantiomer of the fluorescent antifungal azoles. Among the 24 *C. glabrata* strains, 10 were relatively susceptible to **1**_*S*_ (MIC ≤ 4 μg/mL), 10 were moderately resistant (8 μg/mL ≤ MIC ≤ 32 μg/mL), and 4 were highly resistant (MIC ≥ 64). Within the 17 *C. parapsilosis* strains, 14 were susceptible (MIC ≤ 0.5 μg/mL), and 3 exhibited moderate resistance (1 μg/mL ≤ MIC ≤ 4 μg/mL). Similar trends in MIC values were observed for the control azole drug FLC. In agreement with the *in silico* analysis, the antifungal activity of **1**_*R*_ was considerably lower than that of **1**_*S*_.

Both enantiomers exhibited *in vitro* antifungal activity and a resistance spectrum comparable to FLC. The antifungal activity of **1**_*S*_ and **1**_*R*_ was tested against *C. albicans* SN152 and the double knockout strain *erg11ΔΔ/erg3ΔΔ*, which lacks the target Cyp51 and was derived from *C. albicans* SN152 (strains 9 and 15, respectively; Table S1). Both **1**_*S*_ and **1**_*R*_ displayed no activity against the double knockout strain, confirming that like other azole antifungals, Cyp51 is the target of these two enantiomeric antifungal azole probes. Probes **1**_*S*_ and **1**_*R*_ displayed antifungal activities against the parent strain *C. albicans* SN152; however, their potencies, as judged from the MIC values, differed significantly (0.03125 and 4 μg/mL, respectively, Table S4). These results further validate the fluorescent antifungal azole probes **1**_*S*_ and **1**_*R*_ as representatives of the azole class of antifungals and support the *in silico* prediction that the latter is a less potent inhibitor of the target.

### Optimizing Live-Cell Imaging to Explore Azole Probe Uptake and Subcellular Distribution in Live *Candida* Yeast Cells

**Figure 3:**
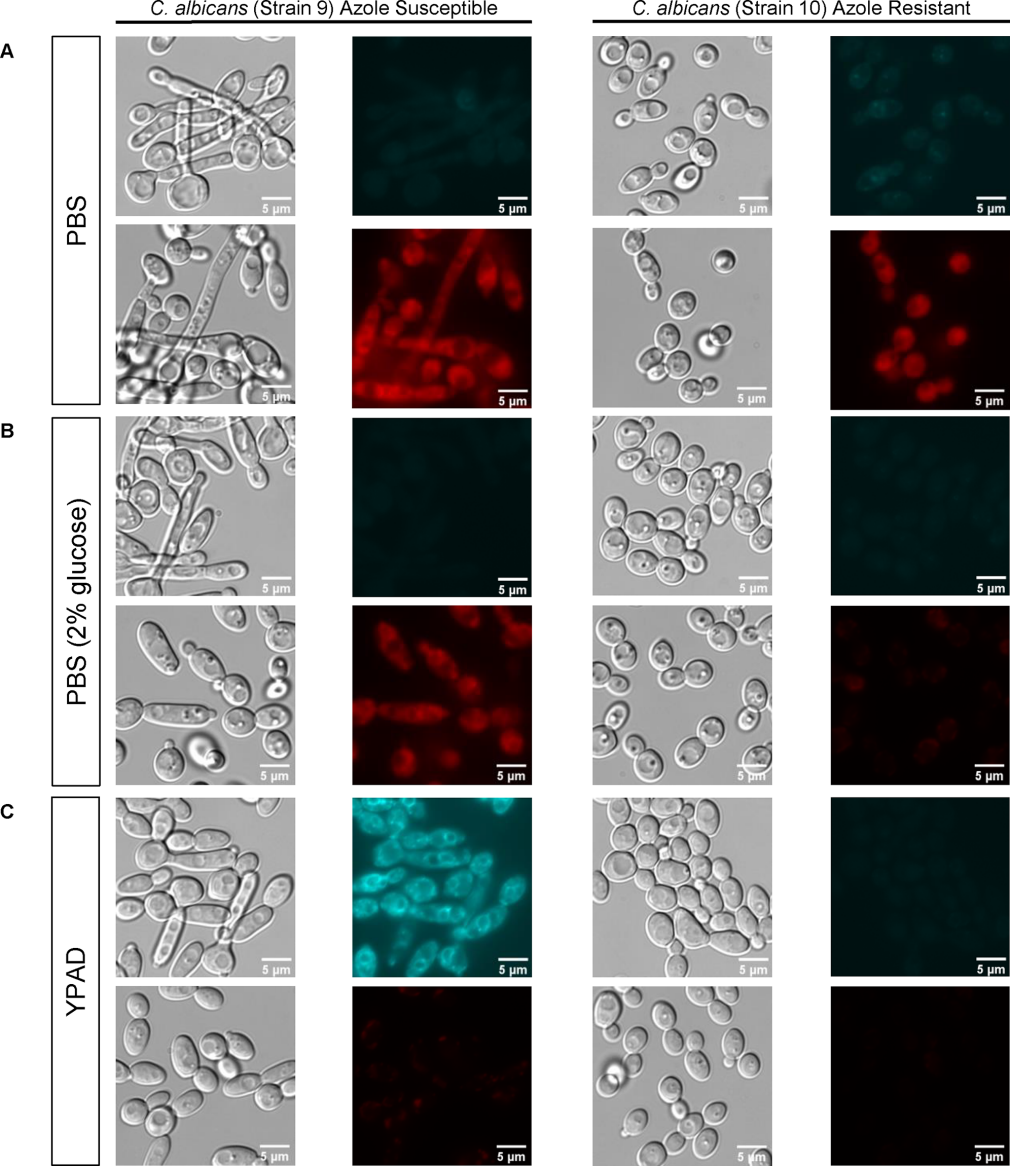
Representative DIC and fluorescent images of yeast cells of the azole-susceptible *C. albicans* SN152 (strain 9) and of the azole-resistant efflux pump expressing *C. albicans* DSY296 (strain 10) incubated with probes **1**_*S*_ or rhodamine 6G in (A) PBS, (B) PBS + 2% glucose, and (C) YPAD. Cells were incubated with probe **1**_*S*_ (1 μM, cyan) or rhodamine 6G (10 μM, red) for 2 h. Scale bars: 5 μm. Images of **1**_*S*_ were collected using a CFP filter (excitation 435/20, emission 480/30), 30% laser intensity, and 2 s exposure. Images of rhodamine 6G were collected using an mCherry filter (excitation 560/40 and emission 635/60), 30% laser intensity, and 500 ms exposure.

While developing the live-cell microscopy procedure, we conducted an optimization of the method to capture live-cell fluorescent images of fluorescent azole probes in the three species of *Candida* represented in the panel. This optimization process involved considerations such as selecting appropriate growth media, determining the minimal detectable concentration of the fluorescent probe, establishing the incubation time prior to live-cell microscopy, and refining the sample preparation process (Figure 3 and Figure S2). The optimization process was conducted on two *C. albicans* strains: the azole-susceptible *C. albicans* SN152 (MIC of probe **1**_*S*_ = 0.03125 μg/mL, strain 9 in Table S1) and the azole-resistant *C. albicans* DSY296 (MIC of probe **1**_*S*_ ≥ 64 μg/mL, strain 10 in Table S1). The latter strain exhibits a high expression of CDR1 and CDR2 efflux pumps. To obtain clear fluorescent images of labeled yeast cells, we selected the minimal detectable probe concentration (1 μM) and an incubation time of 2 h. We also managed to obtain detectable staining with 10 μM of the probe after 1 h of incubation. However, after a 2 h incubation with 10 μM of the probe, fluorescence intensity saturation was witnessed. Thus, the probe **1**_*S*_ concentration of 10 μM does not allow prolonged experiments due to extensive cell damage caused by the antifungal activity of the fluorescent azole probe at this concentration (Figure S2).

No significant labeling with azole probe **1**_*S*_ was observed in both the azole-susceptible and azole-resistant *C. albicans* yeast cells after a 2 h incubation in nutrient-free PBS (Figure 3A, upper panel). In contrast, under the same conditions, the rhodamine 6G fluorescent dye, commonly used to determine the transport activity of yeast membrane efflux pumps, non-selectively labeled both the azole-susceptible and azole-resistant yeast cells (Figure 3A, lower panel). When uptake was evaluated in yeast cells incubated with either azole probe **1**_*S*_ or rhodamine 6G in PBS containing 2% glucose, which facilitates efflux activity, no significant labeling with **1**_*S*_ was observed in the azole-susceptible and azole-resistant yeast cells (Figure 3B, upper panel). In contrast, whereas yeast cells of the azole-susceptible *C. albicans* strain 9 were labeled with rhodamine 6G, no labeling of the cells of the azole-resistant strain 10 was observed (Figure 3B, lower panel).

The nutrient-rich YPAD medium emerged as the most effective medium for monitoring the uptake of the azole probe **1**_*S*_. In this rich medium, ergosterol biosynthesis meets the demands of developing and dividing yeast cells, thereby accentuating the inhibitory impact of azole probes on ergosterol biosynthesis. Notably, the incubation of yeast cells from the azole-susceptible *C. albicans* strain 9 in YPAD enhanced the uptake of azole probe **1**_*S*_ compared to incubation in nutrient-free PBS or in PBS containing 2% glucose (Figure 3C, upper panel). As expected, no significant staining with fluorescent azole probe **1**_*S*_ was observed in cells of the azole-resistant *C. albicans* strain 10 (Figure 3C, upper panel). These results demonstrate that the uptake of fluorescent-based antifungal azole probe **1**_*S*_, which shares the same mode of action as members of the azole class of antifungal drugs, is achievable in a nutrient-rich medium that promotes fungal cell growth and proliferation.

Notably, when yeast cells were incubated with rhodamine 6G in YPAD, no labeling with this fluorescent dye was observed in cells of both *C. albicans* SN152 (strain 9) and *C. albicans* DSY296 (strains 9 and 10, respectively; lower panel in Figure 3C). Presumably, components in the highly nutrient-rich YPAD broth may interfere with the fluorescence or reduce the free fraction of rhodamine 6G, thereby impeding the efficacy of this fluorescent dye-based efflux assay in yeast cell growth-promoting rich media that is required for antifungal azoles to exert their inhibitory effect on ergosterol biosynthesis. Finally, the conditions that proved optimal for fluorescent live-cell imaging of the azole probe **1**_*S*_ were found to be similar for probe **1**_*R*_ (Figure S3).

To conclude, whereas in the case of **1**_*S*_ and **1**_*R*_, cell proliferation, which requires normal ergosterol biosynthesis, is the driving force behind increased intracellular probe accumulation in azole-susceptible cells, in the case of rhodamine 6G, the driving force is likely passive diffusion. Hence, under starvation conditions using PBS, the uptake of rhodamine 6G does not significantly differ between the susceptible and efflux overexpressing strains due to depletion in the levels of ATP necessary for efflux pump activation. When incorporating 2% glucose into the PBS solution, the distinction between the two strain types becomes evident. In this scenario, efflux overexpression significantly influences the outcome. In contrast to rhodamine 6G, in the nutrient-rich medium (YPAD), both antifungal azole probes **1**_*S*_ and **1**_*R*_ accumulate in azole-susceptible yeast cells, displaying subcellular distribution characteristic of the endoplasmic reticulum (ER). These results highlight that the fluorescent azole probes **1**_*S*_ and **1**_*R*_ readily accumulate in azole-susceptible yeast cells in rich media that promote proliferation. Additionally, the uptake of these azoles in high-efflux strains conferring azole resistance is reduced, setting the stage for diagnosing azole resistance resulting from efflux activity.

### The Fluorescent Antifungal Azole Probe **1**_*S*_ Can Detect Azole-Resistant *Candida* Strains That Are Resistant Due to Efflux Pumps

Following the optimized live-cell fluorescence microscopy procedure, we created a database comprised of live-cell images collected from yeast cell samples that were preincubated with the fluorescent azole antifungal probe **1**_*S*_ across the panel of 60 *C. albicans*, *C. glabrata*, and *C. parapsilosis* strains (Database links appear in the Live-Cell Image Analysis part of the Materials and Methods section.). The probe’s uptake was determined by analyzing and averaging six representative images collected from two independent experiments for each strain. Cell and background noise identification was carried out using Nikon NIS Elements’ software thresholding function.

**Figure 4:**
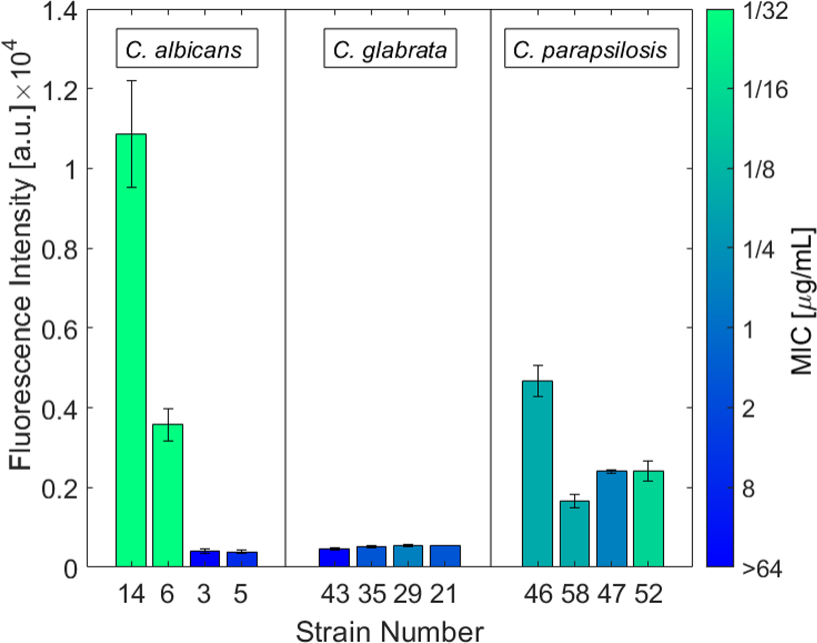
Average cell fluorescence intensity of strains stained with probe **1**_*S*_ (1 μM) plotted against the corresponding MIC values of **1**_*S*_ for these strains. Data are shown for *C. albicans* (strains 14, 6, 3, and 5), *C. glabrata* (strains 43, 35, 29, and 21), and *C. parapsilosis* (strains 46, 58, 47, and 52). MIC values are represented by a gradient color bar. Fluorescence intensity values are presented as means ± SEM.

Exploration of the potential association between the antifungal activity potency of probe **1**_*S*_, determined by its MIC values, and its uptake, represented as the fluorescent intensity in the yeast cells, revealed no discernible correlation (Figure 4).

In some cases, strains exhibiting the same MIC value for **1**_*S*_, displayed substantial differences in the uptake of this probe (e.g., *C. albicans* strains 6 and 14, Figure 4). Conversely, some strains exhibited significantly different MIC values but had a narrow range of uptake levels. For the *C. glabrata* strains in the panel, a narrow distribution of uptake levels was observed despite a wide range of MIC values (e.g., *C. glabrata* strains 21, 29, 35, and 43; Figure 4). Similar trends were observed among the *C. parapsilosis* strains in the panel, where strains with close or even identical MIC values (e.g., *C. parapsilosis* strains 46 and 58, Figure 4) exhibited significant differences in uptake levels. On the other hand, some strains with different MIC values showed similar uptake levels (e.g., *C. parapsilosis* strains 47 and 52, Figure 4). These results indicate that no direct correlation can be established between the azole uptake and its antifungal potency.

**Figure 5:**
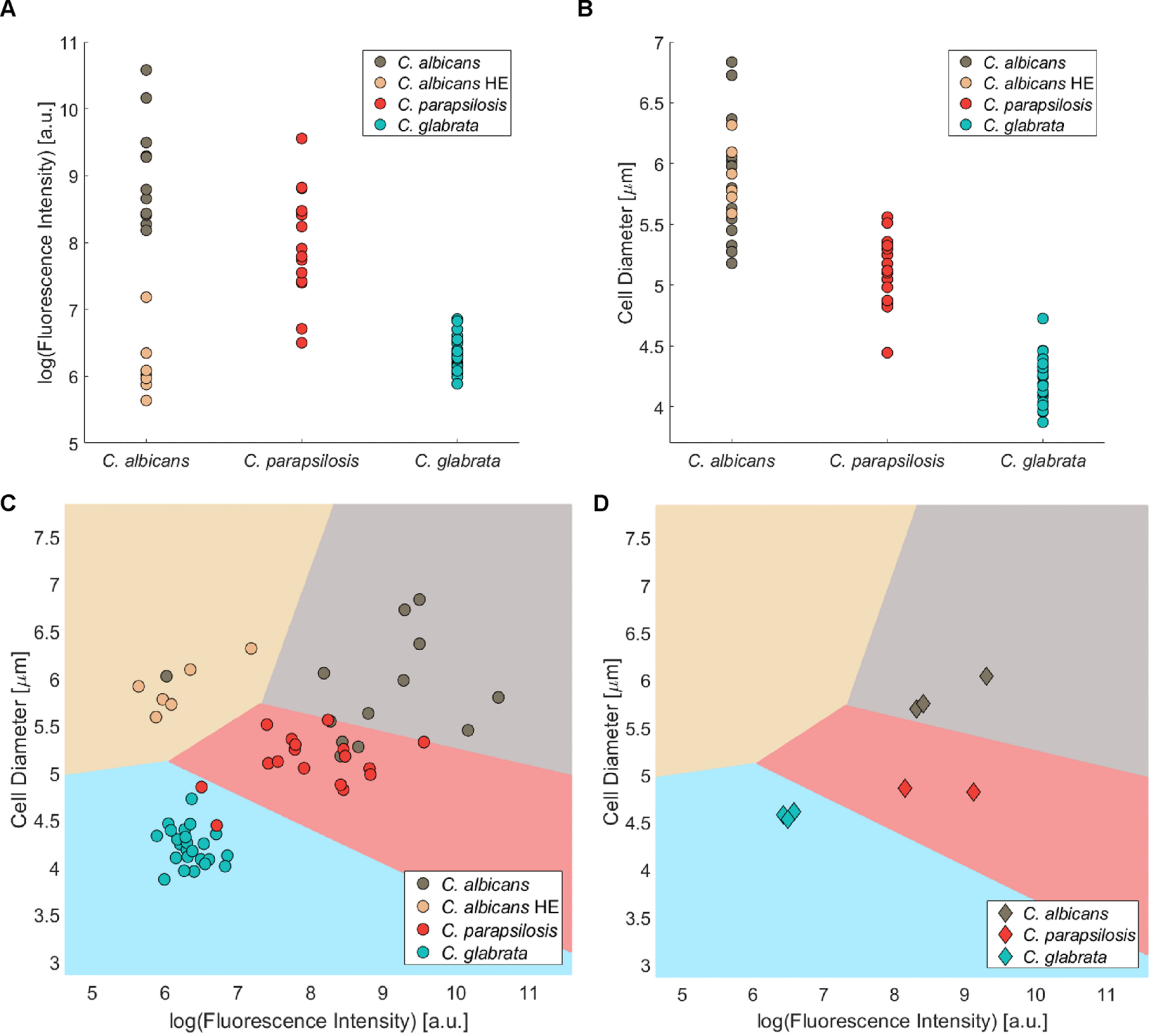
Probe **1**_*S*_ can be used to distinguish fungal species. (A) Distribution of the fluorescence intensity, presented as log(fluorescence intensity), of probe **1**_*S*_ in the panel of *Candida* strains. (B) Distribution of the major cell diameter in the panel of *Candida* strains. (C) Distribution of log(fluorescence intensity) and major yeast cell diameter of the panel of *Candida* strains, classified by a linear discriminant analysis classifier into four distinct populations: *C. albicans*, *C. glabrata*, *C. parapsilosis*, and high efflux (HE)*C. albicans*. A detailed depiction of panel C with error bars is presented in the Supporting Information (Figure S5). (D) Classification of eight *Candida* strains not included in the original panel according to the method presented in panel C (strains V1–V8, Table S7 in the Supporting Information).

Examining the fluorescence intensity values resulting from the cellular uptake of probe **1**_*S*_ for each of the *Candida* species revealed that, although each of the three species exhibited a defined uptake range, considerable overlaps were observed (Figure 5A). For example, among the 19 *C. albicans* strains in the panel, the fluorescence intensity values of nine strains fall within the range of the values measured for the *C. parapsilosis* strains in the panel (Figure 5A). Furthermore, some*C. albicans* strains displaying high efflux, as demonstrated by their genetic background or rhodamine 6G efflux assay (Figure S4), fall within the fluorescence intensity range of the 24 *C. glabrata* strains in the panel (Figure 5A). Consequently, this demonstrated that the uptake of probe **1**_*S*_ into yeast cells, as reflected by fluorescence intensity, cannot serve as a singular descriptor for *Candida* species identification at a high level of confidence.

In seeking to enhance the resolution of the identification process by incorporating an additional descriptor, our attention was directed to differences in the yeast cell sizes among the three *Candida* species. The measured major axis diameter of the yeast cells of *C. albicans* strains ranged between 5.2 and 6.8 μm, that of *C. parapsilosis* ranged between 4.4 and 5.6 μm, and that of *C. glabrata* ranged between 3.9 and 4.7 μm (Figure 5B). However, the major axis diameter ranges measured in the panel revealed a notable overlap between the *C. albicans* strains and the *C. parapsilosis* strains, with the major axis diameters of seven *C. parapsilosis* strains falling within the range of *C. albicans*. Additionally, using this feature, azole-susceptible *C. albicans* strains and HE azole resistant*C. albicans*strains become indistinguishable, as they belong to the same species and share the same yeast cell major axis diameter range.

Interestingly, upon plotting the data acquired from both major axis diameters and fluorescence intensity values of the uptake probe **1**_*S*_, a notable enhancement in the resolution between the four distinguishable populations of strains emerged: *C. albicans*, *C. albicans* HE, *C. glabrata*, and *C. parapsilosis* (Figure 5C). This analysis led to a significant increase in the overall precision of classification to a median of ∼87% for the most suitable classifier compared to ∼73 and ∼75% for intensity or cell diameter classification sorting, respectively (Figure S6).

Finally, to test whether the diagnostic method developed can be used to designate unknown strains, we measured the uptake of fluorescent probe **1**_*S*_ and the large cell diameter of three *C. albicans* strains, three *C. glabrata* strains, and two *C. parapsilosis* strains that were not included in the original panel of 60 strains and applied our identification protocol (Table S7). The results are summarized in Figure 5D. All eight strains were correctly identified as their respective *Candida* species based on the measured fluorescence intensity and large yeast cell diameter descriptors. Moreover, among the three *C. albicans* strains used in this test, all were susceptible to azoles (Table S8); none of these strains were designated as azole-resistant *C. albicans* strains. These results demonstrate the potential utility of the method for providing classification and characterization information.

### Comparing the Uptake of the More Potent Antifungal Fluorescent Probe Enantiomer **1**_*S*_ with the Moderately Potent Enantiomer **1**_*R*_ Reveals the Time Required to Achieve Substantial Cell Permeabilization

**Figure 6:**
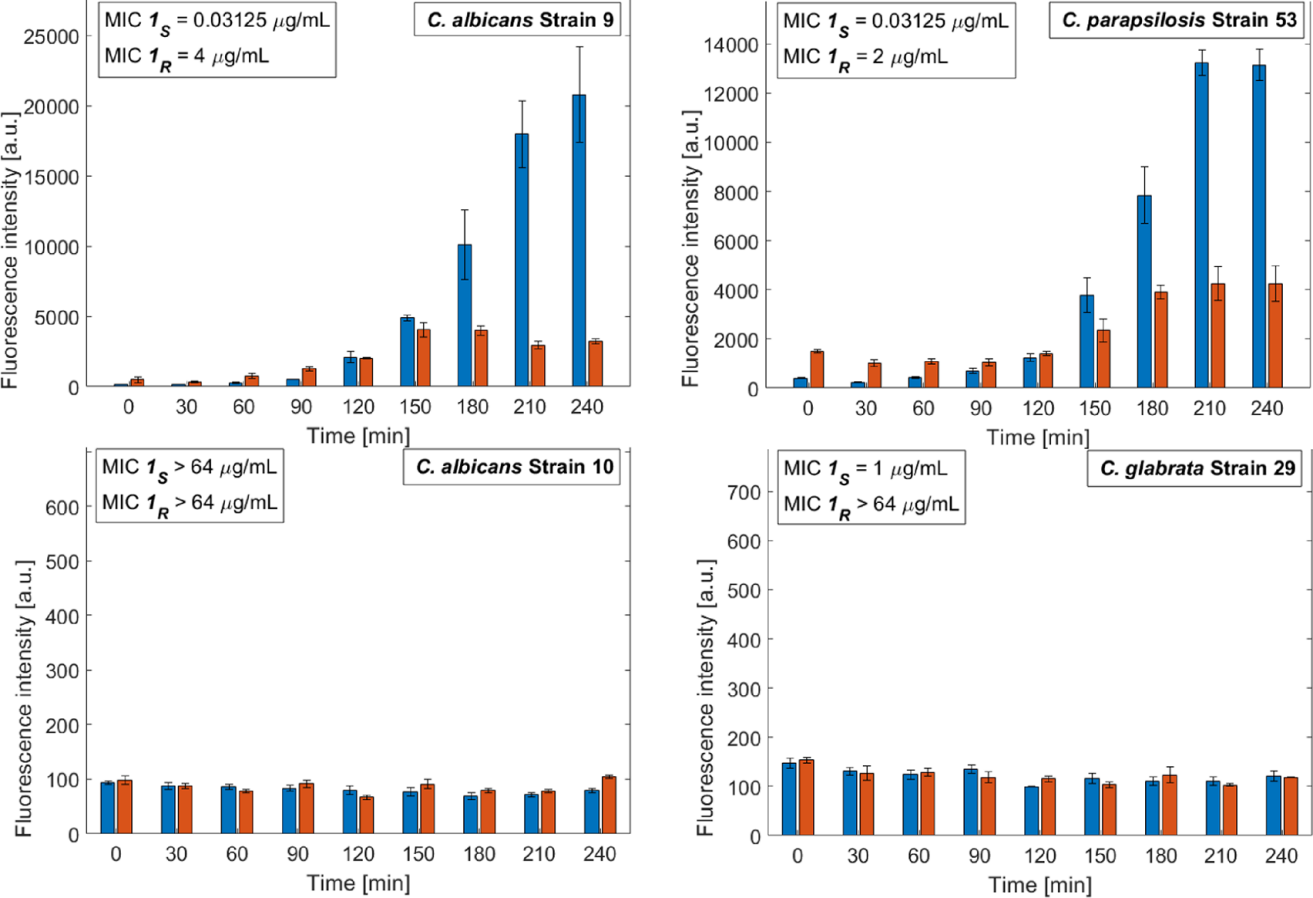
Comparison of time-dependent uptake profiles of probes **1**_*S*_ and **1**_*R*_. Blue bars represent the uptake of probe **1**_*S*_, and orange bars represent the uptake of probe **1**_*R*_. Top left panel: *C. albicans* strain 9; top right panel: *C. parapsilosis* strain 53; bottom left panel: *C. albicans* HE strain 10; bottom right panel: *C. glabrata* strain 29. Fluorescent images were recorded over a 4 h incubation period of the yeast strains with the fluorescent azole probes (1 μM). Results are presented as means ± SEM.

Access to the inherently fluorescent azole antifungal probe enantiomer **1**_*S*_, exhibiting potent *in vitro* antifungal activity, alongside its enantiomer **1**_*R*_, which demonstrates moderate activity, has provided an opportunity to investigate the time frame during which an azole influences membrane permeability in yeast cells across different *Candida* species and to explore whether this effect can be correlated with the antifungal activity potency of the azole. The accumulation profiles of **1**_*S*_ and **1**_*R*_ were investigated over time in two *C. albicans* strains, a *C. parapsilosis* strain, and a *C. glabrata* strain using live-cell microscopy image analysis, and the results are summarized in Figure 6.

The data facilitated the evaluation of the exposure time necessary to observe a substantial increase in the uptake of probe **1**_*S*_ into yeast cells relative to the uptake of **1**_*R*_ as indicated by the fluorescence intensity. We reasoned that this increased uptake is indicative of enhanced membrane permeability attributed to the inhibition in ergosterol biosynthesis relative to the effect of the moderately potent antifungal probe **1**_*R*_. Notably, a divergence in the fluorescence intensities of probes **1**_*S*_ and **1**_*R*_ was observed in the two azole-susceptible strains after approximately 150 min of exposure (*C. albicans* strain 9 and *C. parapsilosis* strain 53, left and right upper panels, respectively, Figure 6). In these two strains, the uptake of moderately active azole probe **1**_*R*_ reached a plateau after approximately 150 min, whereas a sharp increase in the accumulation of the potent enantiomer **1**_*S*_ was observed after 150 min. This result can be attributed to heightened cell permeability, likely caused by a depletion in ergosterol production, which destabilizes the plasma membrane, enhances its permeability, and is more efficient for enantiomer **1**_*S*_.^45^ In the case of the azole-resistant *C. albicans* strain 10 and the azole-resistant *C. glabrata* strain 29, there was a low accumulation of both enantiomers **1**_*S*_ and **1**_*R*_ that did not significantly change throughout the duration of the experiment (left and right lower panels, respectively, Figure 6). Because **1**_*S*_ and **1**_*R*_ are ineffective against these azole-resistant strains, they have little impact on cell membrane permeabilization. As a result, there is minimal change in the accumulation of the probes in the cells of these strains over time.

### Variations in the Subcellular Distribution Patterns of the Fluorescent Azole Probe among *Candida* Species

**Figure 7:**
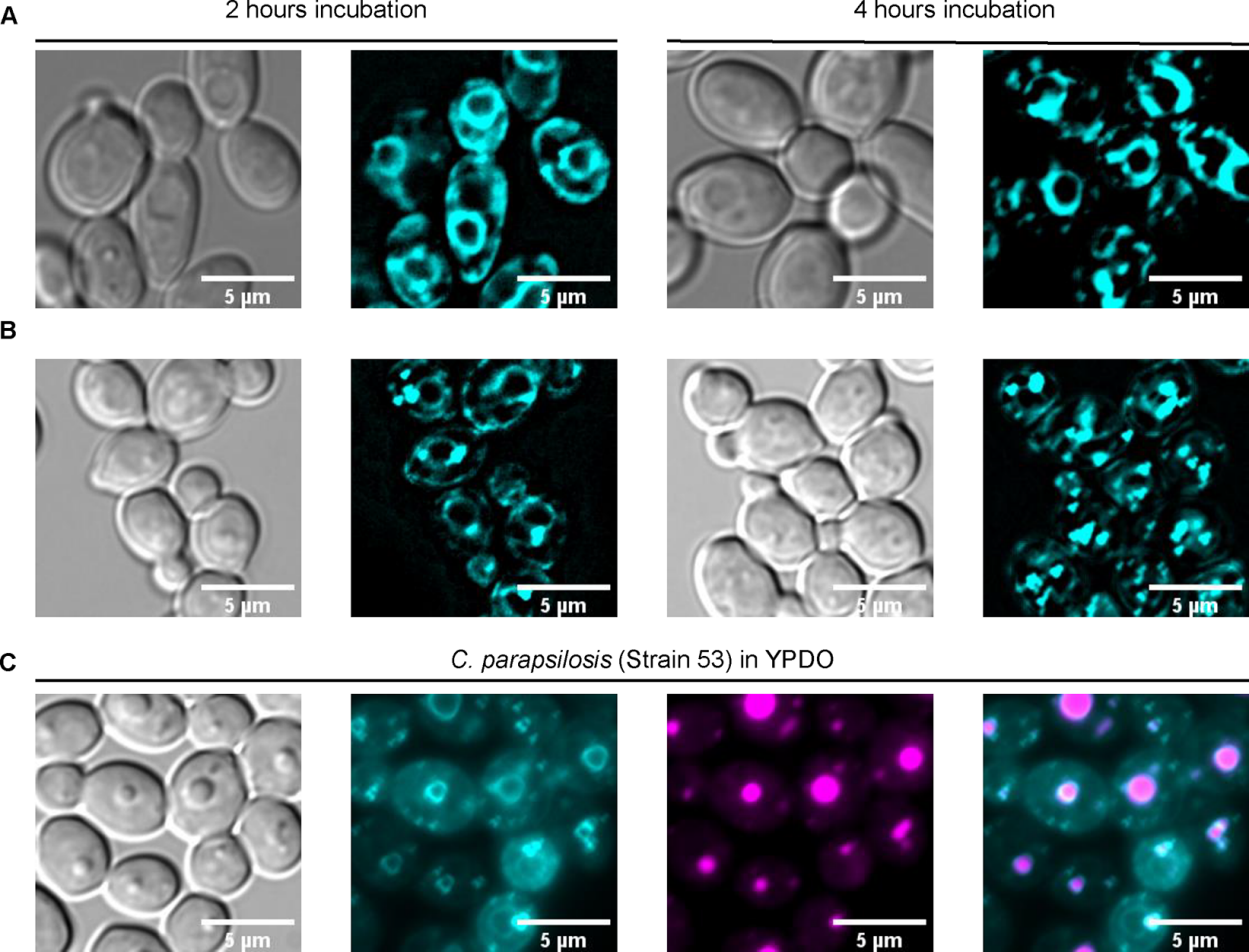
Subcellular distribution of fluorescent azole probe **1**_*S*_ varies between species. (A) *C. albicans* (strain 9, Table S1) and (B) *C. parapsilosis* (strain 53, Table S3). (C) Subcellular distribution of fluorescent azole probe **1**_*S*_ and the lipid droplet tracker pentamethyl BODIPY in *C. parapsilosis* (strain 53, Table S3) grown in YPDO media. Cells were treated with probe **1**_*S*_ (1 μM, cyan) for the 4 h duration of the experiment in panels A and B. Processed fluorescence microscopy images are displayed in panels A and B. The raw images and their processing procedures are shown in Figure S7. In panel C, cells were treated with probe **1**_S_ (1 μM, cyan) for 2 h and pentamethyl BODIPY (0.5 μM, falsely stained in magenta) for 30 min. A merged image of both fluorescent dyes is presented on the right end of panel C. Scale bars represent 5 μm for all images.

We have previously demonstrated that 7-diethyl-aminocoumarin- and BODIPY-based antifungal azole probes primarily localize to the endoplasmic reticulum (ER) in strains of *C. albicans* and *C. glabrata*.^38^ Interestingly, during the observation of subcellular distribution patterns of **1**_*S*_, it became evident that, whereas in all tested *C. albicans* and *C. glabrata* strains treated with **1**_*S*_, mainly the ER was labeled by the fluorescent azole probes (Figure 7A), in all *C. parapsilosis* strains, a notable portion of the fluorescent signal appeared in both the ER and lipid droplets (Figure 7B). Moreover, over the 4 h time-course of live fluorescent microscopy experiments, the ER-characteristic subcellular distribution of **1**_*S*_ remained largely unchanged in the *C. albicans* and *C. glabrata* strains in the panel (Figure 7A). However, in the *C. parapsilosis* strains, the ER-fluorescent pattern gradually weakened, and **1**_*S*_ predominantly accumulated in what appeared as lipid droplets (Figure 7B).

Previous reports on self-protecting strategies of fungi, which involve trapping toxins, have indicated the roles of fungal lipid droplets in drug resistance and adaptations to stress.^46,47^ To further explore the observed change in time dependent delocalization of probe **1**_*S*_ from ER to lipid droplets in *C. parapsilosis*, cells were stained with the fluorescent dye pentamethyl BODIPY, which has been shown to label fungal lipid droplets, in the presence of probe **1**_*S*_.^48−50^ Furthermore, it has been shown that lipid droplets form in fungal cells as a means of protection against glucotoxicity, and their formation can therefore be induced by the addition of glucose. However, this biogenesis is relatively slow, and the maximum droplet size is reached after up to 24 h.^51^ Thus, to investigate the phenomenon further, cell cultures were grown in the presence of 0.1% oleic acid (YPDO media), conditions that rapidly and significantly enlarge lipid droplets in fungal cells.^46^ Under these conditions, after 2 h, larger lipid droplets were formed in *C. parapsilosis* strain 53 (Figure 7C) compared to their formation in YPAD alone.

Interestingly, the increased formation of lipid droplets altered the localization of probe **1**_*S*_ from the ER to the droplets, and the ER staining pattern was lessened in the tested *C. parapsilosis* strain. Of note, the increased size of the lipid droplets facilitated the observation that whereas pentamethyl BODIPY localized inside the lipid droplets, probe **1**_*S*_ mainly localized to the periphery of the lipid droplets in the YPDO-treated *C. parapsilosis* yeast cells (Figure 7C). To further confirm that the organelles stained with probe **1**_*S*_ and/or pentamethyl BODIPY are lipid droplets, cells were further stained with vacuole staining CMAC and nucleus staining DAPI that showed a mismatch between the localization pattern observed for each stain and that of the lipid droplet localizing pentamethyl BODIPY (Figure S8).

Yeast lipid droplets, consisting of a hydrophobic core enveloped by a phospholipid monolayer, exhibit high dynamism, adapting in size, composition, and number in response to cellular conditions. They closely interact with the ER, forming contact sites that facilitate lipid transfer and metabolism.^52−54^ Because azole probe **1**_*S*_ primarily localizes to the ER, the interactions between the ER and lipid droplets likely facilitate the transition of this fluorescent antifungal azole to lipid droplets, especially when their size and number increase. Furthermore, the higher hydrophilicity of probe **1**_*S*_ relative to the pentamethyl BODIPY lipid droplet dye used offers a plausible explanation for its localization to the surface and periphery of the lipid droplets, which are surrounded by the amphiphilic phospholipid layer, whereas pentamethyl BODIPY resides also in the hydrophobic volume of the lipid droplet.

## Conclusions

This study delved into the synthesis and assessment of the *S-* and *R*-enantiomers of a 7-diethylaminocoumarin-based fluorescent antifungal azole probe (**1**_*R*_ and **1**_*S*_). Employing *in silico* analysis, we investigated the interactions of the two enantiomers with target Cyp51. Specifically, **1**_*S*_ interacts with the target binding site via a primary pose with its azole ring interacting with the protein’s heme iron. In contrast, **1**_*R*_ exhibited versatility by adopting two main poses, one involving the azole ring without heme iron interaction, which may explain its moderate *in vitro* antifungal activity compared to that of **1**_*S*_.

A database of fluorescent antifungal azole probe uptake and subcellular distribution images was established and analyzed for a panel of 60 strains belonging to the genus *Candida*, specifically *C. albicans*, *C. glabrata*, and *C. parapsilosis*. This analysis revealed three distinct populations of yeast cells, one for each of the three species represented in the panel, characterized by the correlation between the fluorescent probe uptake and cell diameter. Moreover, differences in uptake were observed between azole-susceptible and azole-resistant strains, with these differences being particularly pronounced in the nutrient-rich broth. This demonstrates the utility of probes **1**_*R*_ and **1**_*S*_ for the rapid diagnosis of azole resistance associated with high efflux or low uptake.

Comparison of the more potent fluorescent antifungal azole probe enantiomer **1**_*S*_ with the moderately potent **1**_*R*_ highlighted differences in the uptake profiles over time. These differences indicate time-dependent membrane-permeabilizing effects required for yeast cell permeabilization and the association with the potency of the antifungal azole. This comparison also highlighted that **1**_*S*_, with its higher antifungal potency compared to **1**_*R*_, causes more substantial damage and fluorescence intensity saturation over time. As a result, **1**_*S*_ is less suitable for longer time-scale localization experiments (over 2 h) than **1**_*R*_.

Live-cell microscopy uncovered unique subcellular distribution patterns, notably seen in *C. parapsilosis* but absent in *C. albicans* or *C. glabrata*. This offers a distinctive strategy involving lipid droplet accumulation, potentially for azole detoxification, introducing an additional diagnostic tool for identifying *C. parapsilosis* based on the time-dependent subcellular distribution pattern of **1**_*R*_ and/or **1**_*S*_.

To conclude, this investigation offers novel insights into the complex dynamic effects of azole antifungals on three clinically important *Candida* species. In addition to contributing to the assessment of antifungal activity, our findings highlight the potential of fluorescent antifungal azole probes as effective tools for species identification and detection of resistance to this crucial class of antifungal drugs.

## Materials and Methods

### General Chemistry Methods and Instrumentation

^1^H NMR spectra were recorded on a Bruker Avance 500 MHz spectrometer. ^13^C NMR spectra were recorded on Bruker Avance 400 and 500 MHz spectrometers at 100 and 125 MHz. Chemical shifts (reported in parts per million) were calibrated to CD_3_OD (^1^H: δ = 3.31, ^13^C: δ = 49.00). Multiplicities are reported using the following abbreviations: s = singlet, d = doublet, t = triplet, dd = doublet of doublets, ddd = doublet of doublets of doublets, m = multiplet. Coupling constants (*J*) are given in hertz. High-resolution electrospray ionization (HRESI) mass spectra were measured on a Waters Xevo G2 XS QTOF instrument. Low-resolution electrospray ionization mass spectrometry (ESI-MS) spectra were measured on a Waters Acquity SQD-2 system. Chemical reactions were monitored by thin-layer chromatography (TLC) (Merck, silica gel 60 F_254_). Visualization was achieved using a cerium molybdate stain (5 g of (NH_4_)_2_Ce(NO_3_)_6_, 120 g of (NH_4_)_6_Mo_7_O_24_·4H_2_O, 80 mL of H_2_SO_4_, 720 mL of H_2_O) or with a UV lamp. All chemicals were obtained from commercial sources. Compounds were purified using Geduran 60 Silica for column chromatography (Merck). The preparative reverse-phase high-pressure liquid chromatography (RP-HPLC) system used was an ECOM system equipped with a 5 μm C-18 Phenomenex Luna Axia column (250 × 21.2 mm). Analytical RP-HPLC was performed on a Hitachi VWR instrument equipped with a diode array detector and an Alltech Apollo C-18 reversed-phase column (5 m, 4.6 × 250 mm). The flow rate was 1 mL/min. Solvent A was 0.1% TFA in water; solvent B was acetonitrile. The SpectraMax i3x Platform spectrophotometer from Molecular Devices was used for fluorescence measurements.

Chiral preparative high-pressure liquid chromatography (HPLC) was performed on an ECOM system equipped with a 5 μm i-Amylose-3 Phenomenex Lux column (250 mm × 21.2 mm). The flow rate was 20 mL/min.

### Synthetic Procedures

Compounds **1**_*S*_ and **1**_*R*_ were synthesized following the synthesis described in Scheme S1 with several modifications:^55^

#### Compound **1**_*S*_

7-Diethylaminocoumarin-3-carboxylic acid (64 mg, 0.24 mmol) was dissolved in dry dimethylformamide (DMF) (2 mL) under argon, treated with HATU (170 mg, 0.45 mmol) and DIPEA (0.14 mL, 0.80 mmol), and stirred at 0 °C for 15 min. Compound 1d-(*S*) (Scheme S1) (47 mg, 0.18 mmol) was added to the reaction mixture and stirred at 0 °C for 15 min. Next, the temperature was allowed to reach the R.T., and the solution was left to stir overnight. Reaction progress was monitored by TLC (petroleum ether/ethyl acetate 30:70). Upon completion, the product was extracted with ethyl acetate and brine. The organic layers were washed with H_2_O (3 × 20 mL), dried over MgSO_4_, and concentrated to obtain the crude product. The product was purified by preparative RP-HPLC (mobile phase: acetonitrile/H_2_O with 0.1% TFA, gradient from 10 to 90% of H_2_O w/ TFA; flow rate: 20 mL/min) to afford 81 mg (88%) of the compound as a yellow powder. HRESI-MS *m*/*z* calcd for C_25_H_25_F_2_N_5_O_4_Na, 520.1772; found [M + Na]+, 520.1780. ^1^H NMR (500 MHz, CD_3_OD) δ 8.52 (s, 1H), 8.36 (s, 1H), 7.78 (s, 1H), 7.51 (m, 1H), 7.47 (d, *J* = 9.0 Hz, 1H), 6.94 (ddd, *J* = 12.4, 9.0, 2.4 Hz, 1H), 6.85 (ddd, *J* = 9.0, 8.3, 2.4 Hz, 1H) 6.77 (dd, *J* = 9.0, 2.4 Hz, 1H), 6.50 (d, *J* = 2.4 Hz, 1H), 4.78 (d, *J* = 14.3 Hz, 1H), 4.67 (d, *J* = 14.3 Hz, 1H), 4.01 (d, *J* = 14.3 Hz, 1H), 3.91 (d, *J* = 14.3 Hz, 1H), 3.50 (q, *J* = 7.1 Hz, 4H), 1.21 (t, *J* = 7.1 Hz, 6H). ^13^C NMR (100 MHz, CD_3_OD) δ 166.2, 165.6, 163.9, 163.1, 162.0, 159.6, 159.2, 154.7, 151.4, 149.4, 146.2, 132.7, 131.4, 125.4, 112.1, 111.7, 109.4, 105.0, 97.2, 76.2, 57.0, 47.6, 46.0, 12.7. ^19^F NMR (376 MHz, CD_3_OD) δ −109.3, −112.9.

#### Compound **1**_*R*_

7-Diethylaminocoumarin-3-carboxylic acid (64 mg, 0.24 mmol) was dissolved in dry dimethylformamide (DMF) (2 mL) under argon, treated with HATU (159 mg, 0.42 mmol) and DIPEA (0.14 mL, 0.80 mmol), and stirred at 0 °C for 15 min. Compound 1d-(*R*) (Scheme S1) (47 mg, 0.18 mmol) was added to the reaction mixture and stirred at 0 °C for 15 min. Next, the temperature was increased to R.T., and the solution was left to stir overnight. Reaction progress was monitored by TLC (petroleum ether/ethyl acetate 30:70). Upon completion, the product was extracted with ethyl acetate and brine. The organic layers were washed with H_2_O (3 × 20 mL), dried over MgSO_4_, and concentrated to obtain the crude product. The product was purified by preparative RP-HPLC (mobile phase: acetonitrile/H_2_O with 0.1% TFA, gradient from 10 to 90% H_2_O w/ TFA; flow rate: 20 mL/min) to afford 77 mg (84%) of the compound as a yellow powder. HRESI-MS *m*/*z* calcd for C_25_H_25_F_2_N_5_O_4_Na, 520.1772; found [M + Na]+, 520.1772. ^1^H NMR (500 MHz, CD_3_OD) δ 8.52 (s, 1H), 8.36 (s, 1H), 7.78 (s, 1H), 7.51 (m, 1H), 7.48 (d, *J* = 9.0 Hz, 1H), 6.95 (ddd, *J* = 12.3, 8.7, 2.4 Hz, 1H), 6.85 (ddd, *J* = 9.3, 8.0, 2.4 Hz, 1H), 6.78 (dd, *J* = 9.0, 2.4 Hz, 1H), 6.51 (d, *J* = 2.4 Hz, 1H), 4.78 (d, *J* = 14.4 Hz, 1H), 4.66 (d, *J* = 14.4 Hz, 1H), 4.02 (d, *J* = 14.1 Hz, 1H), 3.92 (d, *J* = 14.1 Hz, 1H), 3.50 (q, *J* = 7.1 Hz, 4H), 1.21 (t, *J* = 7.1 Hz, 6H). ^13^C NMR (125 MHz, CD_3_OD) δ 166.2, 165.3, 163.9, 163.4, 161.8, 159.8, 159.2, 154.7, 151.4, 149.5, 146.1, 132.7, 131.4, 125.4, 112.1, 111.7, 109.4, 105.0, 97.2, 76.2, 57.0, 47.5, 46.0, 12.7. ^19^F NMR (376 MHz, CD_3_OD) δ −109.3, −112.9.

### Preparation of Stock Solutions of the Tested Compounds

For antifungal activity assays, probes **1**_*S*_, **1**_*R*_ and fluconazole were dissolved in DMSO (5 mg/mL). For microscopy experiments, probes **1**_*S*_, **1**_*R*_, rhodamine 6G, and CellTracker Blue CMAC were dissolved in DMSO (10 mM); pentamethyl BODIPY was dissolved in DMSO (5 mM); and DAPI was dissolved in ddH_2_O (3.6 mM). Fluconazole, rhodamine 6G, and pentamethyl BODIPY were purchased from Sigma-Aldrich. CellTracker Blue CMAC and DAPI were purchased from Thermofisher.

### Minimal Inhibitory Concentration (MIC) Broth Double-Dilution Assay

*C. albicans*, *C. glabrata*, and *C. parapsilosis* MICs were determined using CLSI M27-A3 guidelines with minor modifications. Starter cultures were streaked from a glycerol stock onto YPAD agar plates and grown for 24 h at 30 °C. Several colonies were suspended in 1 mL of PBS and diluted to 1 × 10^–3^ (OD_600_) and then further diluted 1:100 into a fresh medium. Compounds dissolved in DMSO were added to the YPAD broth (32 μL of stock solution in 1218 μL of YPAD broth), and serial double dilutions of the tested compounds in YPAD were prepared in flat-bottomed 96-well microplates (Corning) to enable the testing of concentrations ranging from 64 to 0.015625 μg/mL. Control no-compound wells with yeast cells and blank wells containing only YPAD were prepared. An equal volume (100 μL) of yeast suspensions in YPAD broth was added to each well with the exceptions of the blank wells. MIC values (Tables S4–S6 and S8) were determined after 24 h at 30 °C by observing the growth inside the wells. MIC values were defined as the point at which there is no visible growth compared to the no test compound containing wells. Each concentration was tested in triplicate, and the results were confirmed by two independent sets of experiments. Fluconazole (FLC) was used as a control antifungal azole drug.

### Live-Cell Imaging System Specification

Cells were imaged on a Nikon Ti2 microscope equipped with a Plan Apo λ 100× Oil objective and a Prime BSI A21H204007 camera using the NIS Elements Ar software. The band-pass filter sets used to image rhodamine 6G had an excitation wavelength of 560/40 nm and an emission wavelength of 635/60 nm. For imaging probes **1**_*S*_ and **1**_*R*_, the excitation wavelength was 435/20 nm, and the emission wavelength was 480/30 nm. For pentamethyl BODIPY, the excitation wavelength was 480/30 nm, and the emission wavelength was 535/40 nm. DAPI and CMAC were imaged with an excitation wavelength of 375/28 nm and an emission wavelength of 460/50 nm.

### Live-Cell Imaging

*Candida* strains were streaked from glycerol stocks onto YPAD agar plates and grown for 24 h at 30 °C. Several colonies were then suspended in 3 mL of YPAD broth and grown overnight at 30 °C with shaking in tubes. Starter cultures were diluted 1:50 and incubated in YPAD broth for 2 h at 30 °C with shaking until reaching log-phase growth.

For experiments using probe **1**_*S*_ /**1**_*R*_ or rhodamine 6G alone in YPAD, the probes were directly added and further incubated at 30 °C for 2 h. When staining in PBS or PBS + glucose, cultures were centrifuged, washed with PBS buffer, and resuspended in PBS or PBS + glucose with the desired probe for another 2 h. Stock solutions of probes **1**_*S*_ and **1**_*R*_ were added up to a final concentration of 1 μM, and rhodamine 6G was added up to a final concentration of 10 μM.

For dual staining experiments of probe **1**_*S*_ with pentamethyl BODIPY or CMAC, probe **1**_*S*_ was added directly to the chosen media, and pentamethyl BODIPY/CMAC were added to the broth 30 min before the end of incubation. For dual staining with DAPI, probe **1**_*S*_ was added directly to the chosen media, and upon completion of the incubation, cultures were centrifuged, washed with PBS buffer, and resuspended in PBS buffer with DAPI added for an additional 10 min. For the dual staining experiments, stock solutions of pentamethyl BODIPY, CellTracker Blue CMAC, and DAPI were added up to a final concentration of 0.5, 10, and 36 μM, respectively.

After incubation, cell cultures were centrifuged and washed with 1 mL of PBS buffer until achieving a sufficiently low image background. A 2 μL aliquot of *Candida* cell sample washed with PBS was mounted on a glass slide and covered with a glass coverslip. For qualitative data analysis, experiments conducted over 2 h were illuminated for 2 s at 30% laser intensity, whereas those over 4 h were illuminated for 500 ms at 30% laser intensity. Images were processed by using the NIS Elements Ar software and ImageJ.

### Live-Cell Image Analysis

Databases containing live-cell DIC and corresponding fluorescence microscopy images used for image analysis are available on Mendeley Data. For data used in Figures 4 and 5, please access DOI: 10.17632/g86cb33ck2.1. For data used in Figure 6, please access DOI: 10.17632/hn7p9sm4zv.1. To identify fungal cells for quantitative analysis, the threshold tool in the NIS Elements Ar software was applied to the fluorescent channel to detect yeast cells. Average fluorescent intensities per cell area determined by the software were exported for each image. The mean background intensity of each image was subtracted from the mean cell intensity accordingly. Data from six images across two independent experiments were analyzed, and the final intensity reported is the mean of these images. Model selection for Figures 5A–C was performed using the MATLAB R2021b Classification Learner app with fourfold cross-validation.

### Rhodamine 6G Flow Cytometer Efflux Assay Analysis

*Candida* strains were streaked from glycerol stock onto YPAD agar plates and grown for 24 h at 30 °C. Several colonies were then grown in 3 mL of YPAD broth for 24 h at 30 °C with shaking. The cultures were diluted at 1:50 and incubated in YPAD broth for 2 h at 30 °C with shaking. The cells were washed and resuspended in PBS buffer for 4 h to allow consumption of residual energy sources. A stock solution of rhodamine 6G was added up to a final concentration of 10 μM, and samples were incubated at 30 °C with shaking in the dark for 2 h. Next, samples were washed with PBS and resuspended in a solution of PBS with 2% glucose to begin the experiment. Twenty microliters of the solution was added to 180 μL of PBS in round-bottom 96-well microplates every 10 min for fluorescence intensity measurement throughout an experiment of 50 min duration. Flow cytometry data were collected from at least 10,000 cells per time point using Y1 laser excitation (excitation at 561 nm and emission at 586/15 nm) on an MACSQuant flow cytometer. Analysis was performed using Flowing Software 2.5.1. Results were confirmed by three independent sets of experiments.

### Docking Computations

Molecular docking of CYP51 from *C. albicans* to SKX ligand was carried out in Schrödinger, LLC, 2023, with Glide (Grid-based Ligand Docking with Energetic) XP mode docking methodology.^56^ The crystal 3D structure of CYP51 (Protein Data Bank code 5FSA^41^) was optimized prior to docking using the Protein Preparation Wizard in Schrödinger Maestro Suite 2023 (Schrödinger Suite 2023–4 Protein Preparation Wizard). Inconsistencies in the structure, such as missing side chains or hydrogens, incorrect bond orders, or side-chain orientation, were rectified during optimization,^57^ and the resulting structure was used for docking. Docking of the ligand cannot be performed before the grid generation step. The calculated set of points in a specific grid defines the protein binding site. The ligand, VT1, from the crystal 3D structure of CYP51 (Protein Data Bank code 5TZ1^41^) was superimposed on the 5FSA crystal structure and then used for the binding-site grid generation. The SKX ligand was prepared before docking using the LigPrep module in Schrödinger Maestro Suite 2023 (LigPrep, Schrödinger, 2023–4) to correct impeller bond distances and bond orders, to evaluate ionization states for a given pH, and to generate minimized energy three-dimensional conformations while retaining specified chirality.

The Glide extraprecision (XP) mode weeds out false positives and provides a better correlation between good poses and good scores using a robust sampling protocol. Then, the Prime/MM-GBSA^58^ method based on the docking complex was used to predict the binding-free energy. The molecular mechanics with generalized Born and surface area (MM-GBSA) scoring function has been optimized to predict binding free energies (reported in kcal/mol) for a congeneric series of molecules and is therefore a commonly used follow-up to Glide docking. The reranking of the ligands based on the calculated binding energies (MMGBSA dG Bind) can be expected to agree reasonably well with the ranking based on experimental binding affinity, particularly in the case of congeneric series. As the MM-GBSA binding energies are approximate free energies of binding, a more negative value indicates stronger binding.

### Statistical Analyses

Data are presented as means (two or more replicates) ± SEM (error bars) where applicable. The median prediction accuracy of the classifiers selected in Figure 5C was determined based on histograms depicting the distribution of the overall model prediction accuracy over 10,000 repetitions of training the model using a fourfold cross-validation method. Data analysis was performed in MathWorks MATLAB Version: 9.11.0.2358333 (R2021b) Update 7.

## Supporting Information Available

Demonstration of probes **1**_*S*_ and **1**_*R*_ intramolecular bonds (Figure S1); optimization of probe **1**_*S*_ assay conditions (Figure S2); growth media influence on fluorescent probe **1**_*R*_ uptake (Figure S3); flow cytometry analysis data of high efflux strains (Figure S4); detailed depiction of Figure 5C with its error bars (Figure S5); demonstration of the accuracy of prediction of classifiers selected in Figure 5C (Figure S6); raw live-cell imaging data for Figure 7A,B (Figure S7); subcellular localization of DAPI and CMAC under enlarged droplet conditions (Figure S8); detailed synthetic scheme (Scheme S1); ^1^H, ^13^C and ^19^F NMR spectra (Figures S9–S14); analytic HPLC chromatograms (Figures S15 and S16); absorption and emission spectra (Figures S17 and S18); yeast strains (Tables S1–S3); MIC values (Tables S4–S6); yeast strains used for model validation in Figure 5D (Table S7); and MIC values of strains used for model validation (Table S8) (PDF)

## References

[ref1] Perfect J. R. The Antifungal Pipeline: A Reality Check. Nat. Rev. Drug Discovery 2017 169 2017, 16 (9), 603–616. 10.1038/nrd.2017.46. PMC5760994 28496146

[ref2] Warrilow A. G. S.; Hull C. M.; Parker J. E.; Garvey E. P.; Hoekstra W. J.; Moore W. R.; Schotzinger R. J.; Kelly D. E.; Kelly S. L. The Clinical Candidate VT-1161 Is a Highly Potent Inhibitor of Candida Albicans CYP51 but Fails To Bind the Human Enzyme. Antimicrob. Agents Chemother. 2014, 58 (12), 7121 10.1128/AAC.03707-14. 25224009 PMC4249504

[ref3] Como J. A.; Dismukes W. E. Oral Azole Drugs as Systemic Antifungal Therapy. N. Engl. J. Med. 1994, 330 (4), 263–272. 10.1056/NEJM199401273300407. 8272088

[ref4] Kneale M.; Bartholomew J. S.; Davies E.; Denning D. W. Global Access to Antifungal Therapy and Its Variable Cost. J. Antimicrob. Chemother. 2016, 71 (12), 3599–3606. 10.1093/jac/dkw325. 27516477

[ref5] Vanreppelen G.; Wuyts J.; Van Dijck P.; Vandecruys P. Sources of Antifungal Drugs. J. Fungi 2023, Vol. 9, Page 171 2023, 9 (2), 171 10.3390/jof9020171. PMC9965926 36836286

[ref6] Arastehfar A.; Carvalho A.; Houbraken J.; Lombardi L.; Garcia-Rubio R.; Jenks J. D.; Rivero-Menendez O.; Aljohani R.; Jacobsen I. D.; Berman J.; Osherov N.; Hedayati M. T.; Ilkit M.; Armstrong-James D.; Gabaldón T.; Meletiadis J.; Kostrzewa M.; Pan W.; Lass-Flörl C.; Perlin D. S.; Hoenigl M. Aspergillus Fumigatus and Aspergillosis: From Basics to Clinics. Stud. Mycol. 2021, 100 (1), 100115 10.1016/j.simyco.2021.100115. 34035866 PMC8131930

[ref7] Pereira de Sá N.; Lino C. I.; Fonseca N. C.; Borelli B. M.; Ramos J. P.; Souza-Fagundes E. M.; Rosa C. A.; Santos D. A.; Barbosa De Oliveira R.; Johann S. Thiazole Compounds with Activity against Cryptococcus Gattii and Cryptococcus Neoformans in Vitro. Eur. J. Med. Chem. 2015, 102, 233–242. 10.1016/j.ejmech.2015.07.032. 26276437

[ref8] Netea M. G.; Joosten L. A. B.; Van Der Meer J. W. M.; Kullberg B. J.; Van De Veerdonk F. L. Immune Defence against Candida Fungal Infections. Nat. Rev. Immunol. 2015 1510 2015, 15 (10), 630–642. 10.1038/nri3897. 26388329

[ref9] Patil S.; Rao R. S.; Majumdar B.; Anil S. Clinical Appearance of Oral Candida Infection and Therapeutic Strategies. Front. Microbiol. 2015, 6 (DEC), 167032 10.3389/fmicb.2015.01391. PMC4681845 26733948

[ref10] Pfaller M. A.; Castanheira M. Nosocomial Candidiasis: Antifungal Stewardship and the Importance of Rapid Diagnosis. Med. Mycol. 2015, 54 (1), 1–22. 10.1093/mmy/myv076. 26385381

[ref11] Jørgensen L. N.; Heick T. M. Azole Use in Agriculture, Horticulture, and Wood Preservation – Is It Indispensable?. Front. Cell. Infect. Microbiol. 2021, 11, 730297 10.3389/fcimb.2021.730297. 34557427 PMC8453013

[ref12] Hof H. Critical Annotations to the Use of Azole Antifungals for Plant Protection. Antimicrob. Agents Chemother. 2001, 45 (11), 2987–2990. 10.1128/AAC.45.11.2987-2990.2001. 11600346 PMC90772

[ref13] Nes W. D. Biosynthesis of Cholesterol and Other Sterols. Chem. Rev. 2011, 111 (10), 6423–6451. 10.1021/cr200021m. 21902244 PMC3191736

[ref14] Rodrigues M. L. The Multifunctional Fungal Ergosterol. MBio 2018, 9 (5), 10 10.1128/mBio.01755-18. PMC6143734 30228244

[ref15] Choy H. L.; Gaylord E. A.; Doering T. L. Ergosterol Distribution Controls Surface Structure Formation and Fungal Pathogenicity. MBio 2023, 14, e01353–23. 10.1128/mbio.01353-23. 37409809 PMC10470819

[ref16] Tyndall J. D. A.; Sabherwal M.; Sagatova A. A.; Keniya M. V.; Negroni J.; Wilson R. K.; Woods M. A.; Tietjen K.; Monk B. C. Structural and Functional Elucidation of Yeast Lanosterol 14α-Demethylase in Complex with Agrochemical Antifungals. PLoS One 2016, 11 (12), e0167485 10.1371/journal.pone.0167485. 27907120 PMC5132298

[ref17] Sagatova A. A.; Keniya M. V.; Wilson R. K.; Monk B. C.; Tyndall J. D. A. Structural Insights into Binding of the Antifungal Drug Fluconazole to Saccharomyces Cerevisiae Lanosterol 14-Demethylase. Antimicrob. Agents Chemother. 2015, 59 (8), 4982–4989. 10.1128/AAC.00925-15. 26055382 PMC4505223

[ref18] Perlin D. S.; Rautemaa-Richardson R.; Alastruey-Izquierdo A. The Global Problem of Antifungal Resistance: Prevalence, Mechanisms, and Management. Lancet Infect. Dis. 2017, 17 (12), e383–e392. 10.1016/S1473-3099(17)30316-X. 28774698

[ref19] Berman J.; Krysan D. J. D. R. Drug Resistance and Tolerance in Fungi. Nat. 2020, 18 (6), 319–331. 10.1038/s41579-019-0322-2. PMC7231573 32047294

[ref20] Rautemaa R.; Richardson M.; Pfaller M.; Koukila-Kähkölä P.; Perheentupa J.; Saxén H. Decreased Susceptibility of Candida Albicans to Azole Antifungals: A Complication of Long-Term Treatment in Autoimmune Polyendocrinopathy-Candidiasis-Ectodermal Dystrophy (APECED) Patients. J. Antimicrob. Chemother. 2007, 60 (4), 889–892. 10.1093/jac/dkm299. 17704513

[ref21] Van Dijck P.; Sjollema J.; Camue B. P. A.; Lagrou K.; Berman J.; D’Enfert C.; Andes D. R.; Arendrup M. C.; Brakhage A. A.; Calderone R.; Cantón E.; Coenye T.; Cos P.; Cowen L. E.; Edgerton M.; Espinel-Ingroff A.; Filler S. G.; Ghannoum M.; Gow N. A. R.; Haas H.; Jabra-Rizk M. A.; Johnson E. M.; Lockhart S. R.; Lopez-Ribot J. L.; Maertens J.; Munro C. A.; Nett J. E.; Nobile C. J.; Pfaller M. A.; Ramage G.; Sanglard D.; Sanguinetti M.; Spriet I.; Verweij P. E.; Warris A.; Wauters J.; Yeaman M. R.; Zaat S. A. J.; Thevissen K. Methodologies for in Vitro and in Vivo Evaluation of Efficacy of Antifungal and Antibiofilm Agents and Surface Coatings against Fungal Biofilms. Microb. Cell 2018, 5 (7), 300 10.15698/mic2018.07.638. 29992128 PMC6035839

[ref22] Price C. L.; Parker J. E.; Warrilow A. G.; Kelly D. E.; Kelly S. L. Azole Fungicides – Understanding Resistance Mechanisms in Agricultural Fungal Pathogens. Pest Manag. Sci. 2015, 71 (8), 1054–1058. 10.1002/ps.4029. 25914201

[ref23] Lee Y.; Robbins N.; Cowen L. E. Molecular Mechanisms Governing Antifungal Drug Resistance. *npj Antimicrob*. Resist. 2023 11 2023, 1 (1), 1–9. 10.1038/s44259-023-00007-2. PMC11057204 38686214

[ref24] Siikala E.; Rautemaa R.; Richardson M.; Saxen H.; Bowyer P.; Sanglard D. Persistent Candida Albicans Colonization and Molecular Mechanisms of Azole Resistance in Autoimmune Polyendocrinopathy–Candidiasis–Ectodermal Dystrophy (APECED) Patients. J. Antimicrob. Chemother. 2010, 65 (12), 2505–2513. 10.1093/jac/dkq354. 20876623

[ref25] Ksiezopolska E.; Gabaldón T. Evolutionary Emergence of Drug Resistance in Candida Opportunistic Pathogens. Genes 2018 2018, 9 (9), 461 10.3390/genes9090461. PMC6162425 30235884

[ref26] Ferrari S.; Ischer F.; Calabrese D.; Posteraro B.; Sanguinetti M.; Fadda G.; Rohde B.; Bauser C.; Bader O.; Sanglard D. Gain of Function Mutations in CgPDR1 of Candida Glabrata Not Only Mediate Antifungal Resistance but Also Enhance Virulence. PLOS Pathog. 2009, 5 (1), e1000268 10.1371/journal.ppat.1000268. 19148266 PMC2607542

[ref27] Arastehfar A.; Lass-Flörl C.; Garcia-Rubio R.; Daneshnia F.; Ilkit M.; Boekhout T.; Gabaldon T.; Perlin D. S. The Quiet and Underappreciated Rise of Drug-Resistant Invasive Fungal Pathogens. J. Fungi 2020, 6 (3), 138 10.3390/jof6030138. PMC7557958 32824785

[ref28] Healey K. R.; Perlin D. S. Fungal Resistance to Echinocandins and the MDR Phenomenon in Candida Glabrata. J. Fungi 2018, 4 (3), 105 10.3390/jof4030105. PMC6162769 30200517

[ref29] Arastehfar A.; Kargar M. L.; Mohammadi S. R.; Roudbary M.; Ghods N.; Haghighi L.; Daneshnia F.; Tavakoli M.; Jafarzadeh J.; Hedayati M. T.; Wang H.; Fang W.; Carvalho A.; Ilkit M.; Perlin D. S.; Lass-Flörl C. A High Rate of Recurrent Vulvovaginal Candidiasis and Therapeutic Failure of Azole Derivatives Among Iranian Women. Front. Microbiol. 2021, 12, 655069 10.3389/fmicb.2021.655069. 33995315 PMC8113757

[ref30] Wang Z.; Xing B. Small-Molecule Fluorescent Probes: Big Future for Specific Bacterial Labeling and Infection Detection. Chem. Commun. 2021, 58 (2), 155–170. 10.1039/D1CC05531C. 34882159

[ref31] Shi H.; Kwok R. T. K.; Liu J.; Xing B.; Tang B. Z.; Liu B. Real-Time Monitoring of Cell Apoptosis and Drug Screening Using Fluorescent Light-up Probe with Aggregation-Induced Emission Characteristics. J. Am. Chem. Soc. 2012, 134 (43), 17972–17981. 10.1021/ja3064588. 23043485

[ref32] Sedgwick A. C.; Brewster J. T.; Harvey P.; Iovan D. A.; Smith G.; He X. P.; Tian H.; Sessler J. L.; James T. D. Metal-Based Imaging Agents: Progress towards Interrogating Neurodegenerative Disease. Chem. Soc. Rev. 2020, 49 (10), 2886–2915. 10.1039/C8CS00986D. 32226991

[ref33] Chao D. H. M.; Kallemeijn W. W.; Marques A. R. A.; Orre M.; Ottenhoff R.; Van Roomen C.; Foppen E.; Renner M. C.; Moeton M.; Van Eijk M.; Boot R. G.; Kamphuis W.; Hol E. M.; Aten J.; Overkleeft H. S.; Kalsbeek A.; Aerts J. M. F. G. Visualization of Active Glucocerebrosidase in Rodent Brain with High Spatial Resolution Following In Situ Labeling with Fluorescent Activity Based Probes. PLoS One 2015, 10 (9), e0138107 10.1371/journal.pone.0138107. 26418157 PMC4587854

[ref34] Hoogendoorn S.; Blom A. E. M.; Willems L. I.; Van Der Marel G. A.; Overkleeft H. S. Synthesis of PH-Activatable Red Fluorescent BODIPY Dyes with Distinct Functionalities. Org. Lett. 2011, 13 (20), 5656–5659. 10.1021/ol202379w. 21942639

[ref35] Benhamou R. I.; Bibi M.; Steinbuch K. B.; Engel H.; Levin M.; Roichman Y.; Berman J.; Fridman M. Real-Time Imaging of the Azole Class of Antifungal Drugs in Live Candida Cells. ACS Chem. Biol. 2017, 12 (7), 1769–1777. 10.1021/acschembio.7b00339. 28472585 PMC7030953

[ref36] Lepesheva G. I.; Friggeri L.; Waterman M. R. CYP51 as Drug Targets for Fungi and Protozoan Parasites: Past, Present and Future. Parasitology 2018, 145 (14), 1820–1836. 10.1017/S0031182018000562. 29642960 PMC6185833

[ref37] Roundtree M. T.; Juvvadi P. R.; Shwab E. K.; Cole D. C.; Steinbach W. J. Aspergillus Fumigatus Cyp51A and Cyp51B Proteins Are Compensatory in Function and Localize Differentially in Response to Antifungals and Cell Wall Inhibitors. Antimicrob. Agents Chemother. 2020, 64 (10), e00735-20 10.1128/AAC.00735-20. 32660997 PMC7508596

[ref38] Benhamou R. I.; Jaber Q. Z.; Herzog I. M.; Roichman Y.; Fridman M. Fluorescent Tracking of the Endoplasmic Reticulum in Live Pathogenic Fungal Cells. ACS Chem. Biol. 2018, 13 (12), 3325–3332. 10.1021/acschembio.8b00782. 30427174

[ref39] Elias R.; Benhamou R. I.; Jaber Q. Z.; Dorot O.; Zada S. L.; Oved K.; Pichinuk E.; Fridman M. Antifungal Activity, Mode of Action Variability, and Subcellular Distribution of Coumarin-Based Antifungal Azoles. Eur. J. Med. Chem. 2019, 179, 779–790. 10.1016/j.ejmech.2019.07.003. 31288127

[ref40] Benhamou R. I.; Bibi M.; Berman J.; Fridman M. Localizing Antifungal Drugs to the Correct Organelle Can Markedly Enhance Their Efficacy. Angew. Chem. Int. 2018, 57 (21), 6230–6235. 10.1002/anie.201802509. PMC7035955 29575397

[ref41] Hargrove T. Y.; Friggeri L.; Wawrzak Z.; Qi A.; Hoekstra W. J.; Schotzinger R. J.; York J. D.; Guengerich F. P.; Lepesheva G. I. Structural Analyses of Candida Albicans Sterol 14α-Demethylase Complexed with Azole Drugs Address the Molecular Basis of Azole-Mediated Inhibition of Fungal Sterol Biosynthesis. J. Biol. Chem. 2017, 292 (16), 6728–6743. 10.1074/jbc.M117.778308. 28258218 PMC5399120

[ref42] Brown G. D.; Denning D. W.; Gow N. A. R.; Levitz S. M.; Netea M. G.; White T. C. Hidden Killers: Human Fungal Infections. Sci. Transl. Med. 2012, 4 (165), 165rv13 10.1126/scitranslmed.3004404. 23253612

[ref43] Silva S.; Henriques M.; Martins A.; Oliveira R.; Williams D.; Azeredo J. Biofilms of Non-Candida Albicans Candida Species: Quantification, Structure and Matrix Composition. Med. Mycol. 2009, 47 (7), 681–689. 10.3109/13693780802549594. 19888800

[ref44] Guinea J. Global Trends in the Distribution of Candida Species Causing Candidemia. Clin. Microbiol. Infect. 2014, 20 (6), 5–10. 10.1111/1469-0691.12539. 24506442

[ref45] Zhang Y. Q.; Gamarra S.; Garcia-Effron G.; Park S.; Perlin D. S.; Rao R. Requirement for Ergosterol in V-ATPase Function Underlies Antifungal Activity of Azole Drugs. PLOS Pathog. 2010, 6 (6), e1000939 10.1371/journal.ppat.1000939. 20532216 PMC2880581

[ref46] Chang W.; Zhang M.; Zheng S.; Li Y.; Li X.; Li W.; Li G.; Lin Z.; Xie Z.; Zhao Z.; Lou H. Trapping Toxins within Lipid Droplets Is a Resistance Mechanism in Fungi. Sci. Reports 2015 51 2015, 5 (1), 1–11. 10.1038/srep15133. PMC4604559 26463663

[ref47] Brink J. T. R.; Fourie R.; Sebolai O.; Albertyn J.; Pohl C. H. The Role of Lipid Droplets in Microbial Pathogenesis. J. Med. Microbiol. 2021, 70 (6), 001383 10.1099/jmm.0.001383. 34184983

[ref48] Wang J.; Guo X.; Li L.; Qiu H.; Zhang Z.; Wang Y.; Sun G. Application of the Fluorescent Dye BODIPY in the Study of Lipid Dynamics of the Rice Blast Fungus Magnaporthe Oryzae. Molecules 2018, 23 (7), 1594 10.3390/molecules23071594. 29966327 PMC6099410

[ref49] Kobae Y.; Gutjahr C.; Paszkowski U.; Kojima T.; Fujiwara T.; Hata S. Lipid Droplets of Arbuscular Mycorrhizal Fungi Emerge in Concert with Arbuscule Collapse. Plant Cell Physiol. 2014, 55 (11), 1945–1953. 10.1093/pcp/pcu123. 25231957

[ref50] Li Y.; Zhu J.; Hu J.; Meng X.; Zhang Q.; Zhu K.; Chen X.; Chen X.; Li G.; Wang Z.; Lu G. Functional Characterization of Electron-Transferring Flavoprotein and Its Dehydrogenase Required for Fungal Development and Plant Infection by the Rice Blast Fungus. Sci. Reports 2016 61 2016, 6 (1), 1–13. 10.1038/srep24911. PMC4845064 27113712

[ref51] Nguyen L. N.; Nosanchuk J. D. Lipid Droplet Formation Protects against Gluco/Lipotoxicity in Candida Parapsilosis: An Essential Role of Fatty Acid Desaturase Ole1. Cell Cycle 2011, 10 (18), 3159–3167. 10.4161/cc.10.18.16932. 21897120

[ref52] Hariri H.; Rogers S.; Ugrankar R.; Liu Y. L.; Feathers R.; Henne M. Lipid Droplet Biogenesis Is Spatially Coordinated at ER-Vacuole Contacts under Nutritional Stress. EMBO Rep. 2018, 19 (1), 57–72. 10.15252/embr.201744815. 29146766 PMC5757283

[ref53] Choudhary V.; Schneiter R. A Unique Junctional Interface at Contact Sites Between the Endoplasmic Reticulum and Lipid Droplets. Front. Cell Dev. Biol. 2021, 9, 650186 10.3389/fcell.2021.650186. 33898445 PMC8060488

[ref54] Salo V. T.; Ikonen E. Moving out but Keeping in Touch: Contacts between Endoplasmic Reticulum and Lipid Droplets. Curr. Opin. Cell Biol. 2019, 57, 64–70. 10.1016/j.ceb.2018.11.002. 30476754

[ref55] Benhamou R. I.; Bibi M.; Berman J.; Fridman M. Localizing Antifungal Drugs to the Correct Organelle Can Markedly Enhance Their Efficacy. Angew. Chemie Int. Ed. 2018, 57 (21), 6230–6235. 10.1002/anie.201802509. PMC7035955 29575397

[ref56] Friesner R. A.; Murphy R. B.; Repasky M. P.; Frye L. L.; Greenwood J. R.; Halgren T. A.; Sanschagrin P. C.; Mainz D. T. Extra Precision Glide: Docking and Scoring Incorporating a Model of Hydrophobic Enclosure for Protein-Ligand Complexes. J. Med. Chem. 2006, 49 (21), 6177–6196. 10.1021/jm051256o. 17034125

[ref57] Madhavi Sastry G.; Adzhigirey M.; Day T.; Annabhimoju R.; Sherman W. Protein and Ligand Preparation: Parameters, Protocols, and Influence on Virtual Screening Enrichments. J. Comput. Aided. Mol. Des. 2013, 27 (3), 221–234. 10.1007/s10822-013-9644-8. 23579614

[ref58] Li J.; Abel R.; Zhu K.; Cao Y.; Zhao S.; Friesner R. A. The VSGB 2.0 Model: A next Generation Energy Model for High Resolution Protein Structure Modeling. Proteins 2011, 79 (10), 2794–2812. 10.1002/prot.23106. 21905107 PMC3206729

