# Supplementary material for: Chiral Fluorescent Antifungal Azole Probes Detect Resistance, Uptake Dynamics, and Subcellular Distribution in Candida Species
Source: JACS Au. 2024 Aug 13;4(8):3157–69. doi: 10.1021/jacsau.4c00479 (PMC11350599; doi:10.1021/jacsau.4c00479)
Supplement: Supplementary file 1 — au4c00479_si_001.pdf [file au4c00479_si_001.pdf]

Supporting Information

Chiral Fluorescent Antifungal Azole Probes Detect Resistance, Uptake Dynamics, and Subcellular Distribution in *Candida* Species

Vlad Koren[a], Efrat Ben-Zeev[b], Ivan Voronov[a], Micha Fridman[a,*]

[a] *School of Chemistry, Raymond and Beverley Sackler Faculty of Exact Sciences, Tel Aviv University, Tel Aviv, 6997801, Israel.*

[b] *Ilana and Pascal Mantoux Institute for Bioinformatics and Nancy and Stephen Grand Israel National Center for Personalized Medicine, Weizmann Institute of Science, 7610001 Rehovot, Israel.*

*Correspondence: mfridman@tauex.tau.ac.il

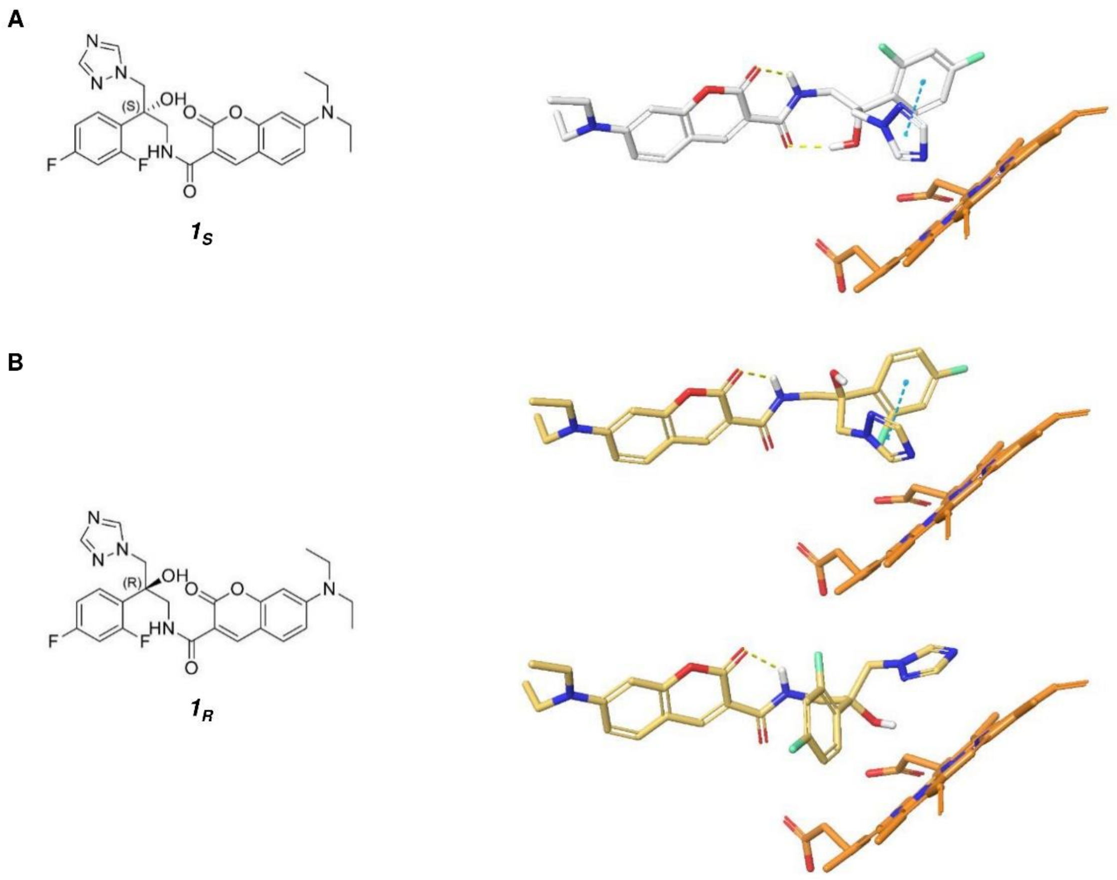

Figure S1. The intramolecular bonds resulting from the 3D geometry of the two enantiomers of the fluorescent antifungal azole probes. A) The $\mathbf{1_S}$ probe exhibits two hydrogen bonds (depicted by yellow dashed lines) and intra-ligand π-π stacking interactions (shown by cyan dashed lines. **B)** Probe $\mathbf{1_R}$, lacks the hydrogen bond between the benzylic tertiary alcohol and the amide carbonyl. Additionally, the π-π stacking interaction between the triazole and difluorobenzene rings is present only in one of the conformations. These results are the top-ranked docking outcomes using the Schrödinger Glide XP docking protocol, with parameters set to save 50 poses per ligand. The ligand (voriconazole) from the structure with PDB code 5FSA was used for grid construction. Flexible MM-GBSA was employed to re-rank the docking results.

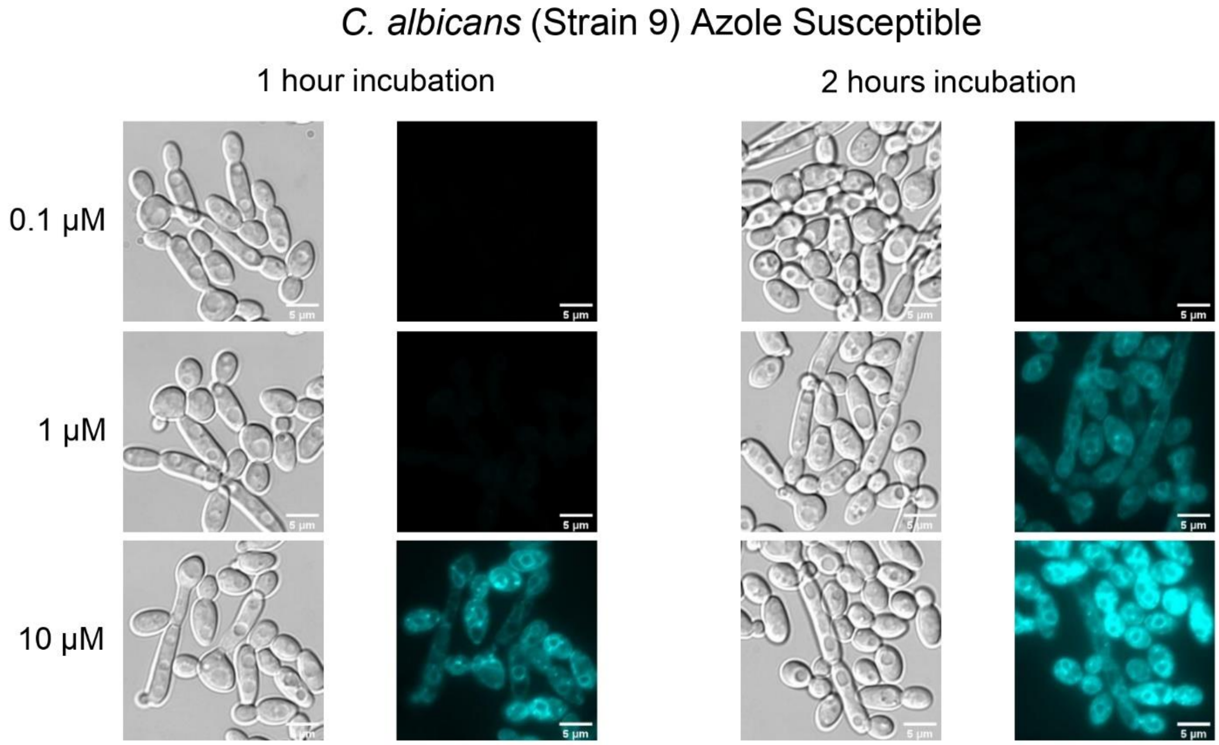

Figure S2. Optimization of concentration and required incubation time of fluorescent probe *1*$_S$ uptake in yeast cells. Representative DIC and fluorescent images of azole susceptible *C. albicans* SN152 incubated with probe ***1***$_S$ (cyan) in YPAD. Scale bars, 5 µm.

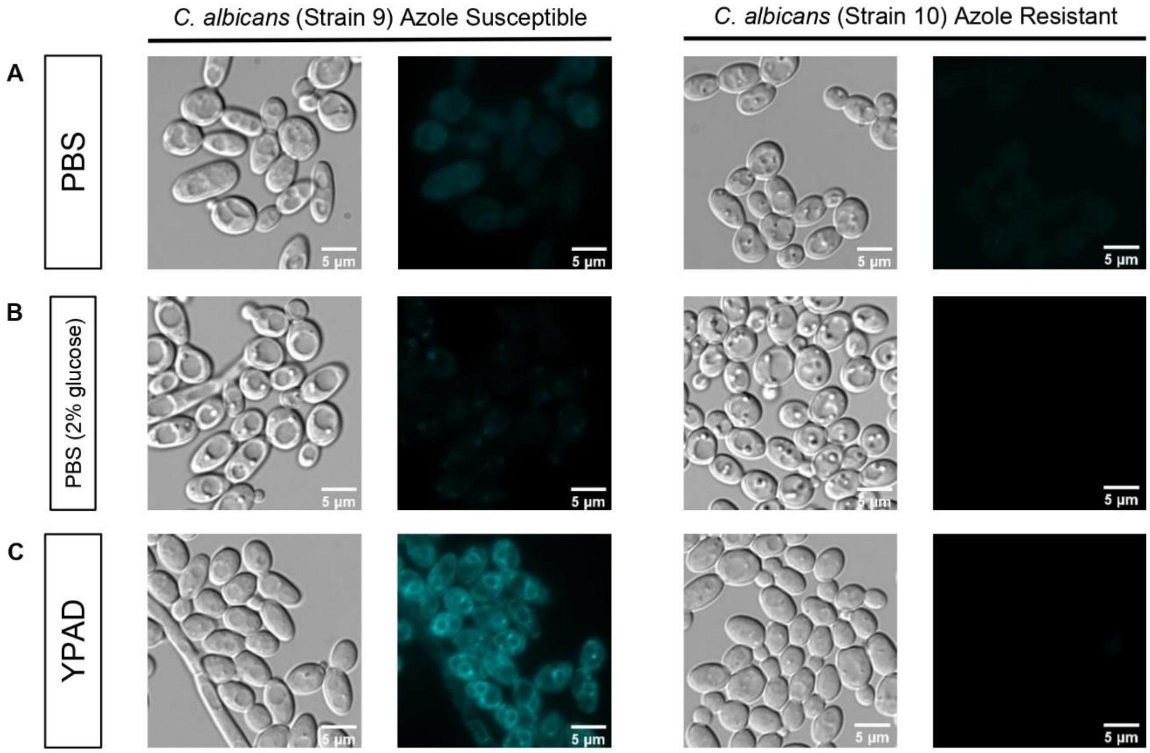

Figure S3. Growth media influence on fluorescent probe *1ᴿ* uptake in yeast cells. Representative DIC and fluorescent images of yeast cells of the azole susceptible *C. albicans* strain 9 and of the azole resistant efflux pump overexpressing *C. albicans* strain 10 incubated with probes ***1_R*** (1 µM, cyan) in: **A)** PBS; **B)** PBS+2% glucose; **C)** YPAD. Cells were incubated with probe ***1_R*** (1 µM, cyan) for 2 hours. Scale bars, 5 µm.

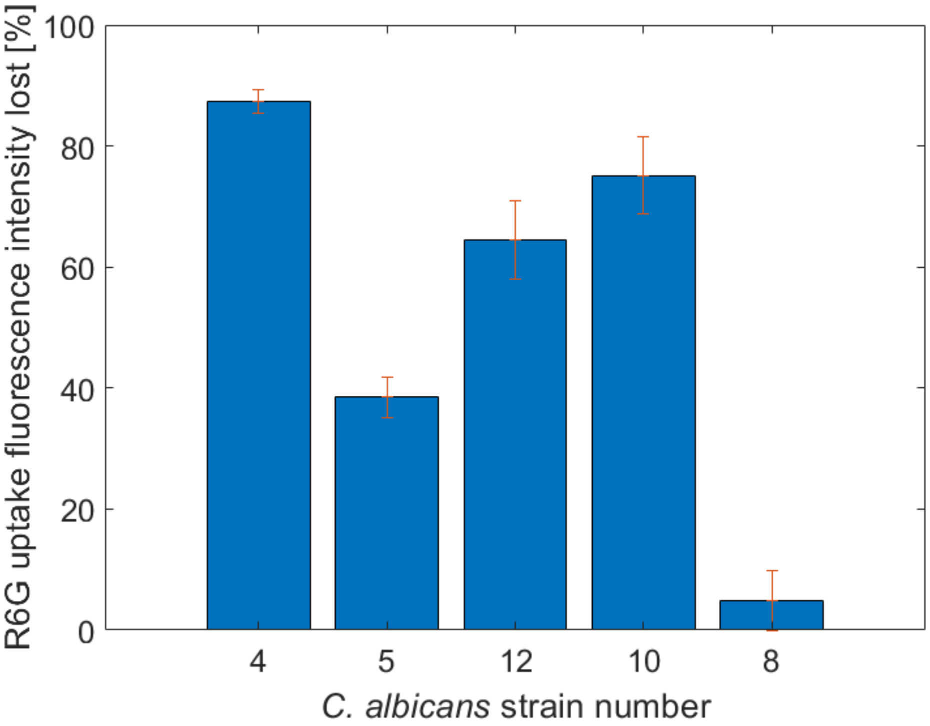

Figure S4. Evaluation of efflux by change in fluorescence intensity of rhodamine 6G (R6G). The uptake of R6G was monitored using flow cytometry over 50 minutes. Strain 10 was genetically characterized as efflux pump overexpressing and therefore used as positive control and strain 8 was used as negative control. Error bars shown as standard error of 3 independent measurements. The intensity loss was calculated according to Equation 1.

Equation 1. $$Intensity\,Loss = 100 \times \frac{I_0 - I_{50}}{I_0}$$

I_0 - Intensity measured at 0 minutes.

I_{50} - Intensity measured at 50 minutes.

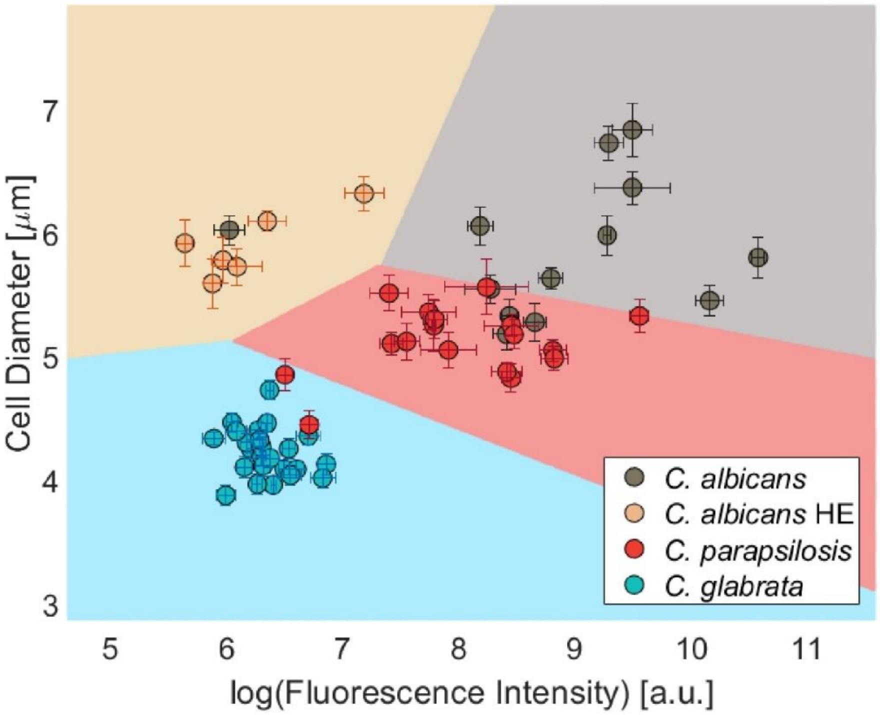

Figure S5. Distribution of log(fluorescence intensity) and major yeast cell diameter. A panel of *Candida* strains was classified by a linear discriminant analysis classifier into four distinct populations: *C. albicans*, *C. glabrata*, *C. parapsilosis*, high efflux *C. albicans*. Error bars are presented as SEM for the vertical axis and calculated according to Equation 2 for the horizontal axis.

Equation 2. $\Delta_x = \frac{SEM_x}{\bar{x}}$

Δ_x - Error of a given point at the horizontal axis on log-scale.

SEM_x - Standard error of the mean of a given point at the horizontal axis on a linear scale.

$\bar{x}$ - The value at a given data point which was computed as the average of 6 images.

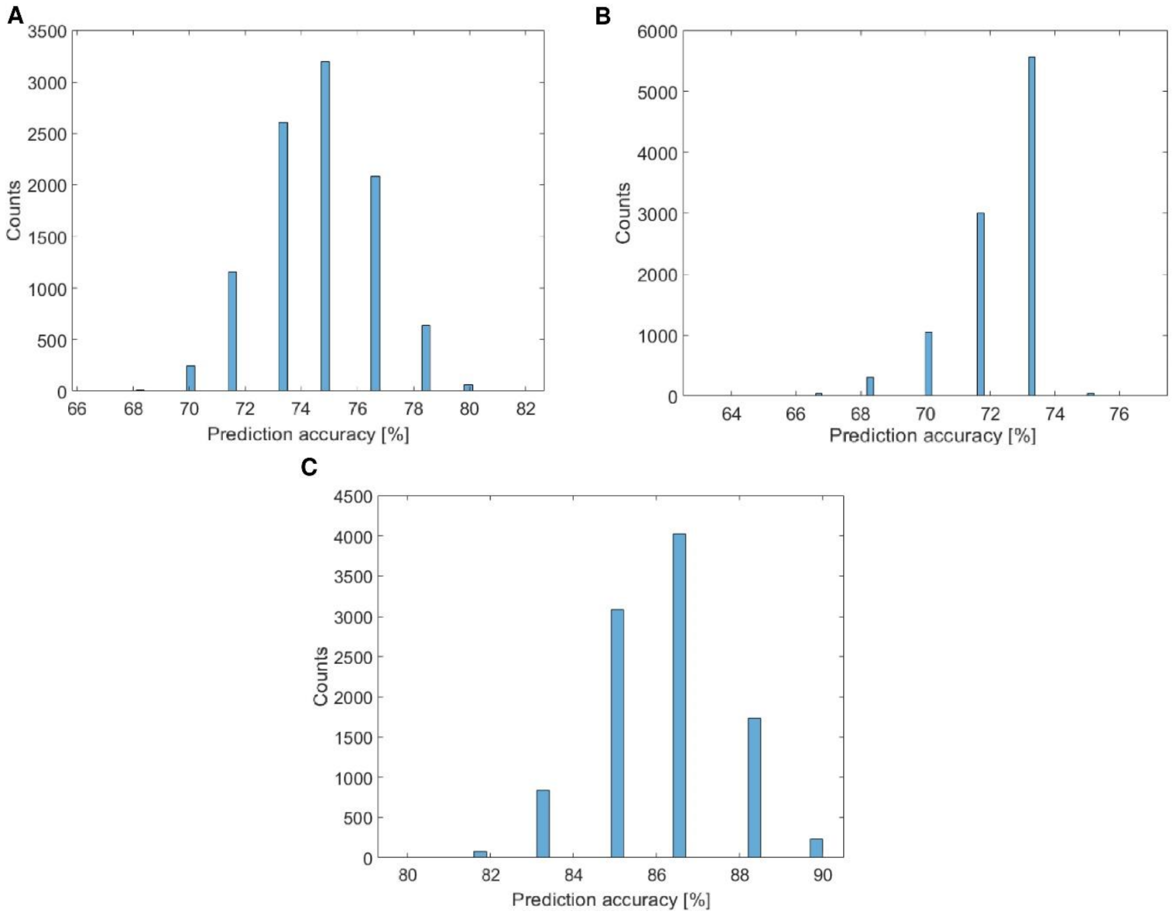

Figure S6. Prediction accuracy of classifiers selected in Figure 4D. Histograms depicting the distribution of the overall model prediction accuracy over 10,000 repetitions of training the model using a 4-fold cross-validation method when the classification is based on: **A)** Major cell diameter; **B)** log(Fluorescence Intensity); **C)** Major cell diameter and log(Fluorescence Intensity).

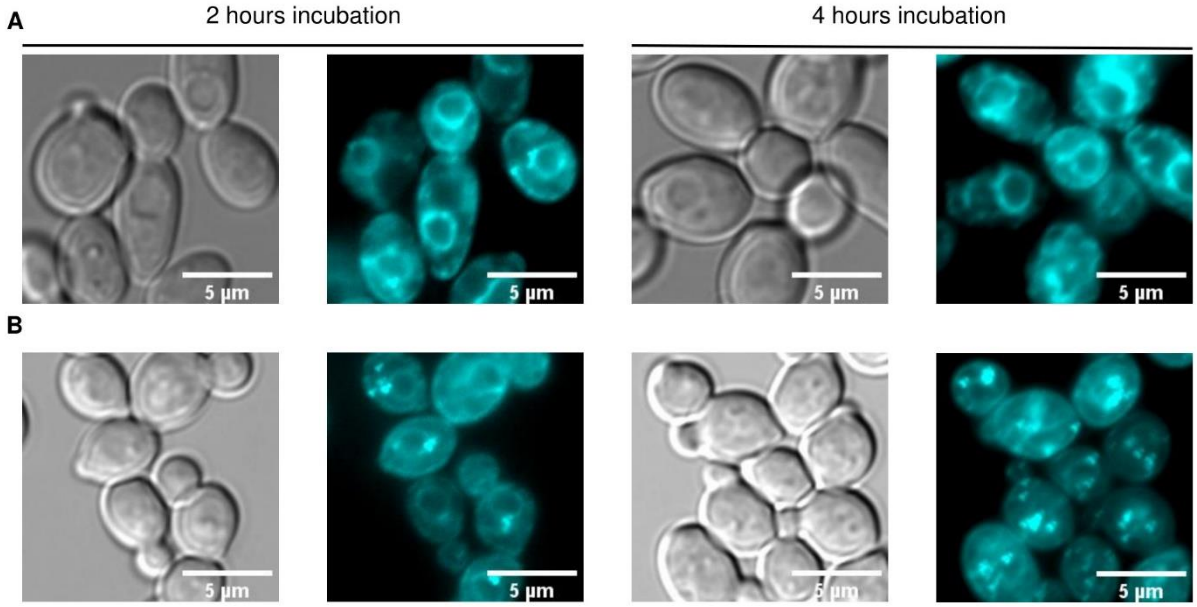

Figure S7. Subcellular distribution of fluorescent azole probe *1s* at two different time points. A) *C. albicans* (strain 9, Table S1), left panel – 2-hour incubation, right panel – 4-hour incubation; **B)** *C. parapsilosis* (strain 53, Table S1), left panel – 2-hour incubation, right panel – 4-hour incubation. Cells were treated with probe ***1s*** (1 µM, cyan) for the entire 4-hour duration. Scale bars, 5 µm. To transform the raw images presented in this figure into the images presented in Figure 6 in the manuscript, images were processed and optimized using NIS Elements AR software. The following sequence was applied: background subtraction based on the mean background of the image, optimization of local contrast, rolling ball correction, and optimization of the median filter.

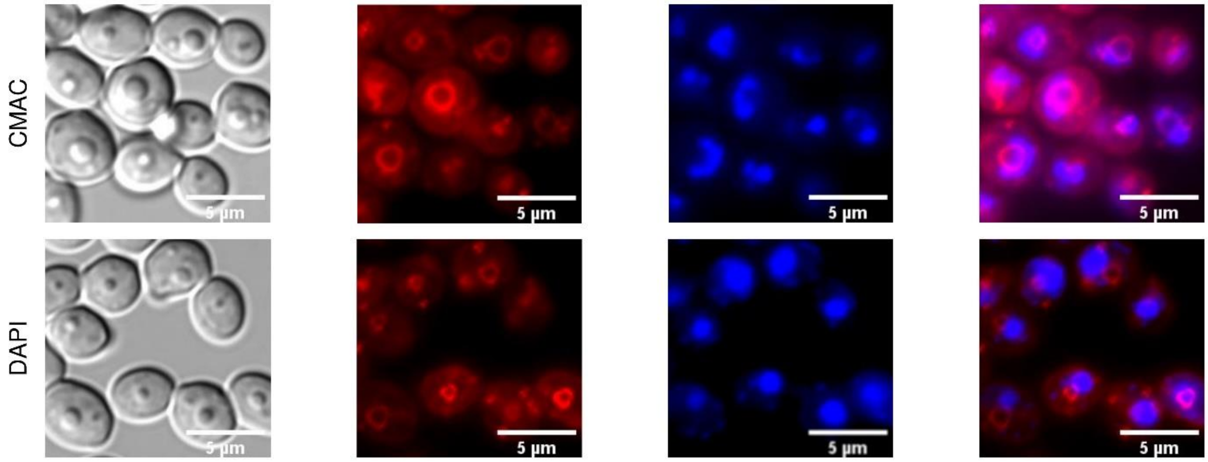

Figure S8. Subcellular distribution of fluorescent azole probe *1s* and vacuole staining CMAC/nucleus staining DAPI. Experiment was performed over a 2-hour incubation in YPDO in *C. parapsilosis* (strain 53, Table S3). Cells were treated with probe ***1s*** (1 µM, falsely stained in red) and CMAC (10 µM, blue) or probe ***1s*** (1 µM, falsely stained in red) and DAPI (36 µM, blue) as stated in the methodology section. Scale bars, 5 µm. The rightmost panel represents a composite image.

Synthetic scheme

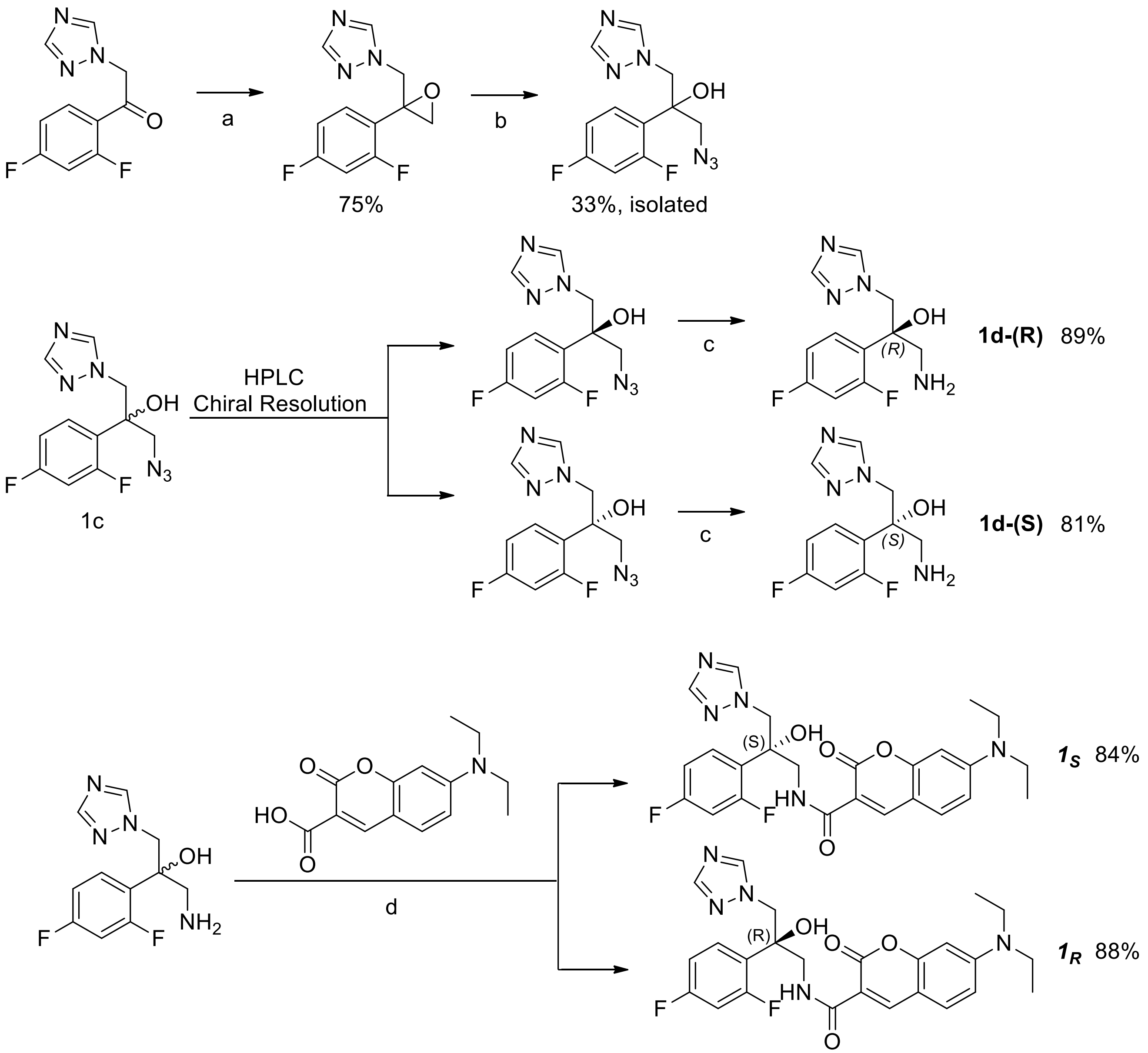

Scheme S1. a) Trimethylsulfoxonium iodide, NaOH 20%, toluene; b) NaN_3, DMF; c) H_2/Pd, isopropanol; d) HATU/DIPEA, DMF.

NMR Spectra

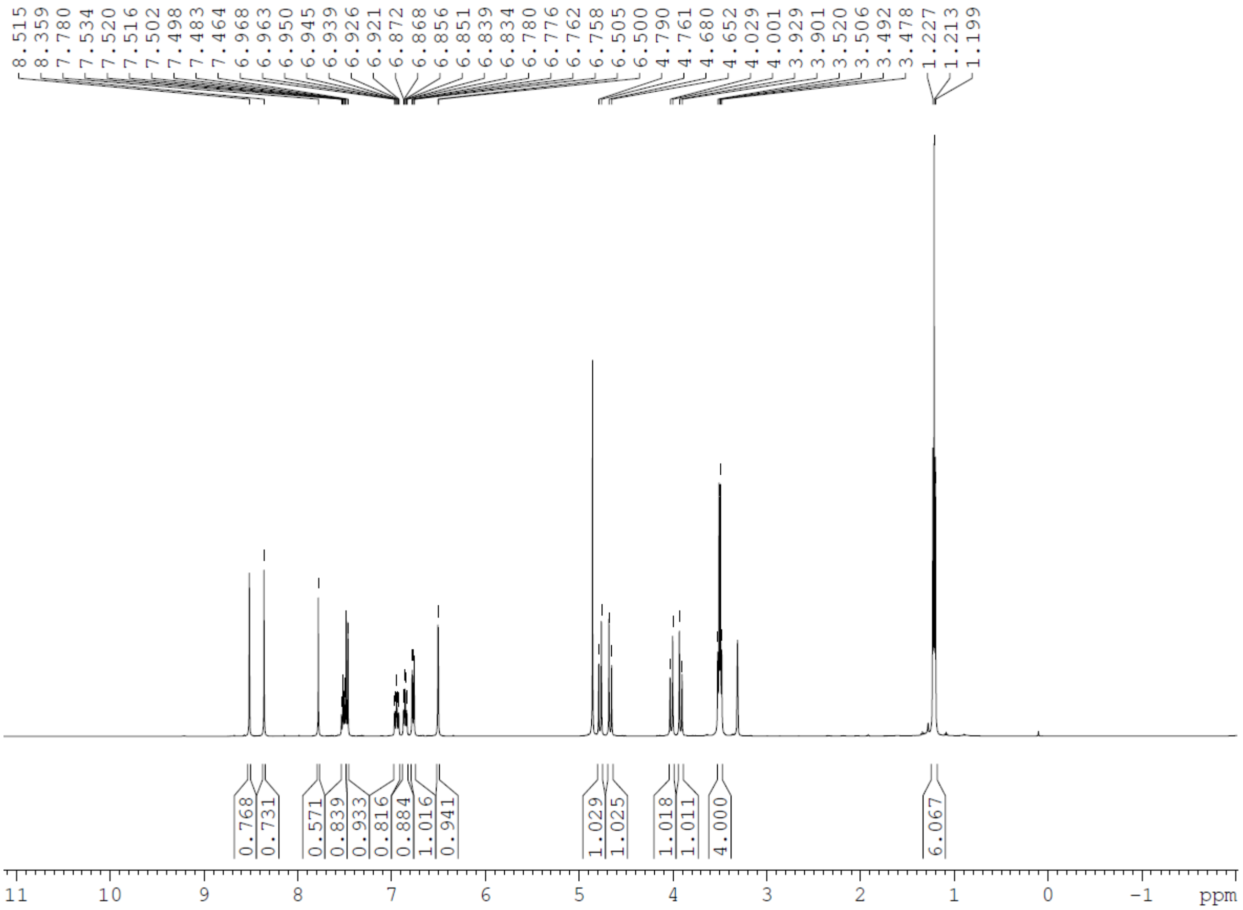

Figure S9. 500 MHz ^{1}H-NMR spectrum of compound ***1s*** in CD_3OD

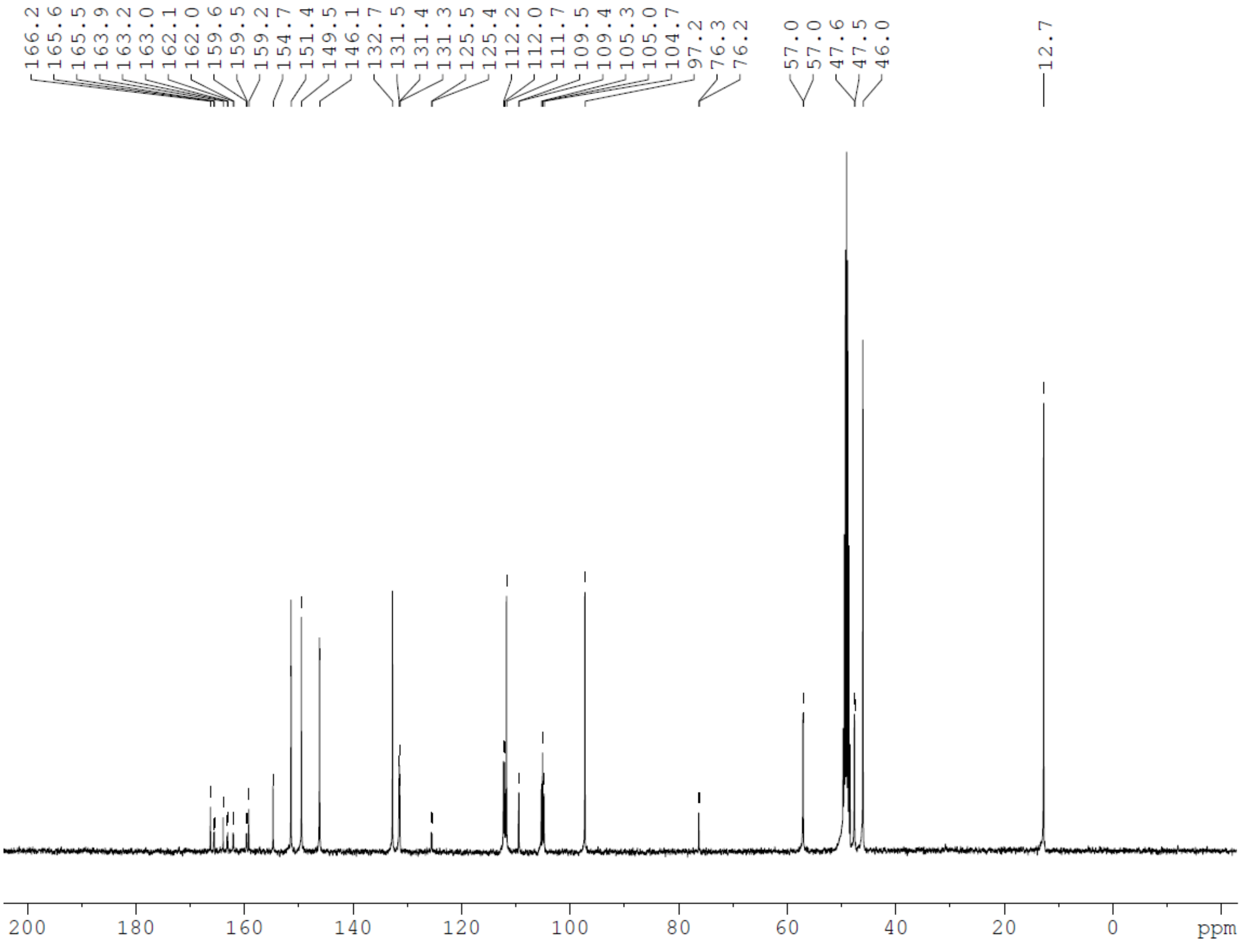

Figure S10. 100 MHz ^{13}C-NMR spectrum of compound ***1s*** in CD_3OD

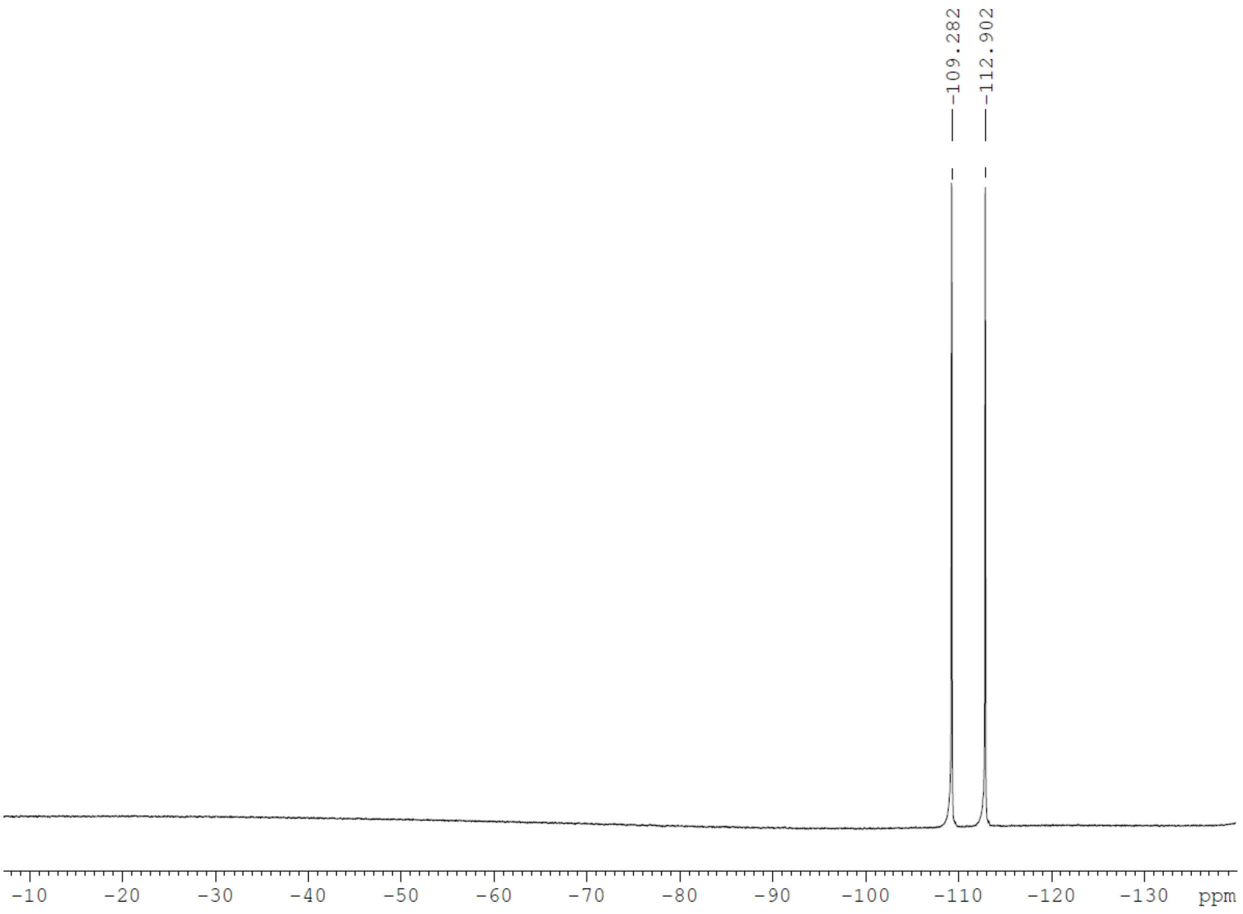

Figure S11. 376 MHz ^{19}F-NMR spectrum of compound ***1s*** in CD_3OD

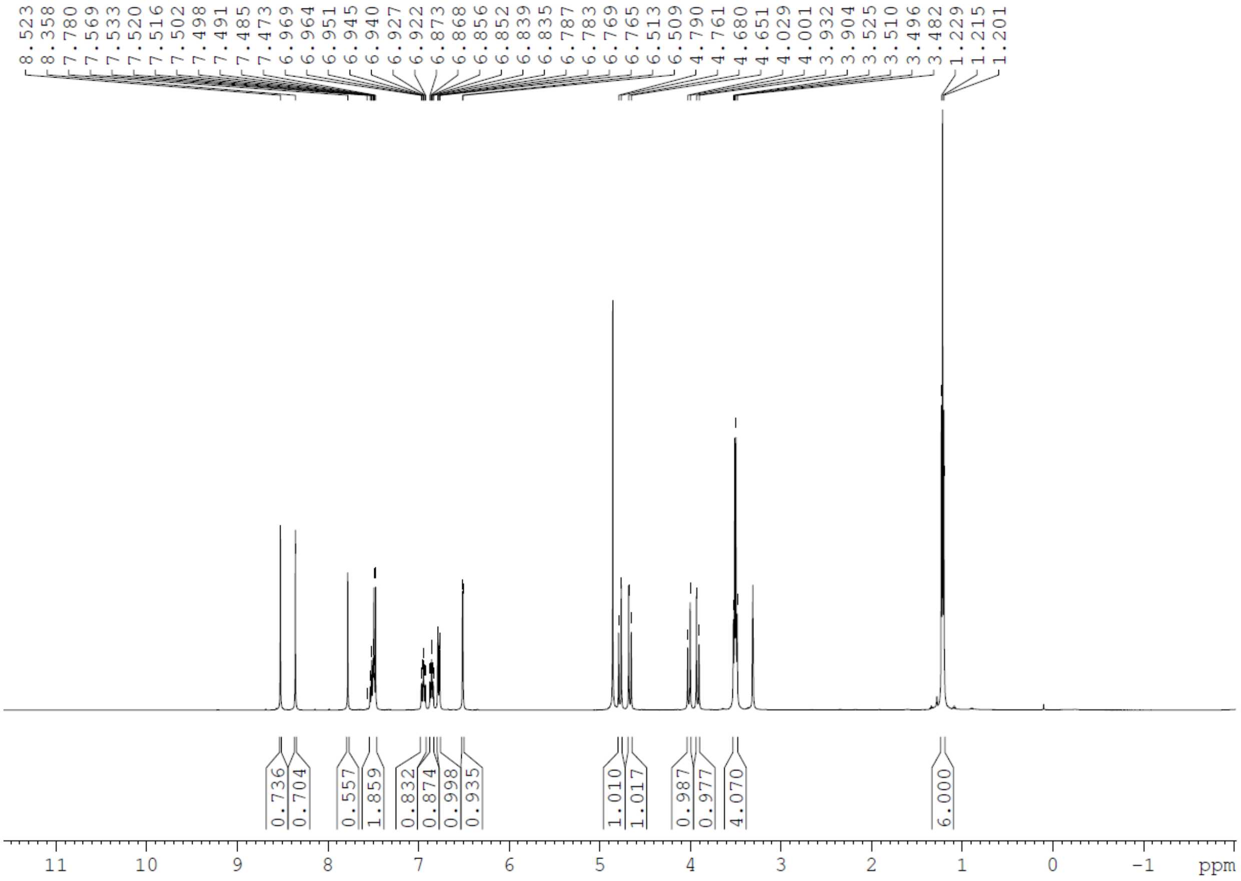

Figure S12. 500 MHz ^{1}H-NMR spectrum of compound ***1*** $_{\boldsymbol{R}}$ in CD_3OD

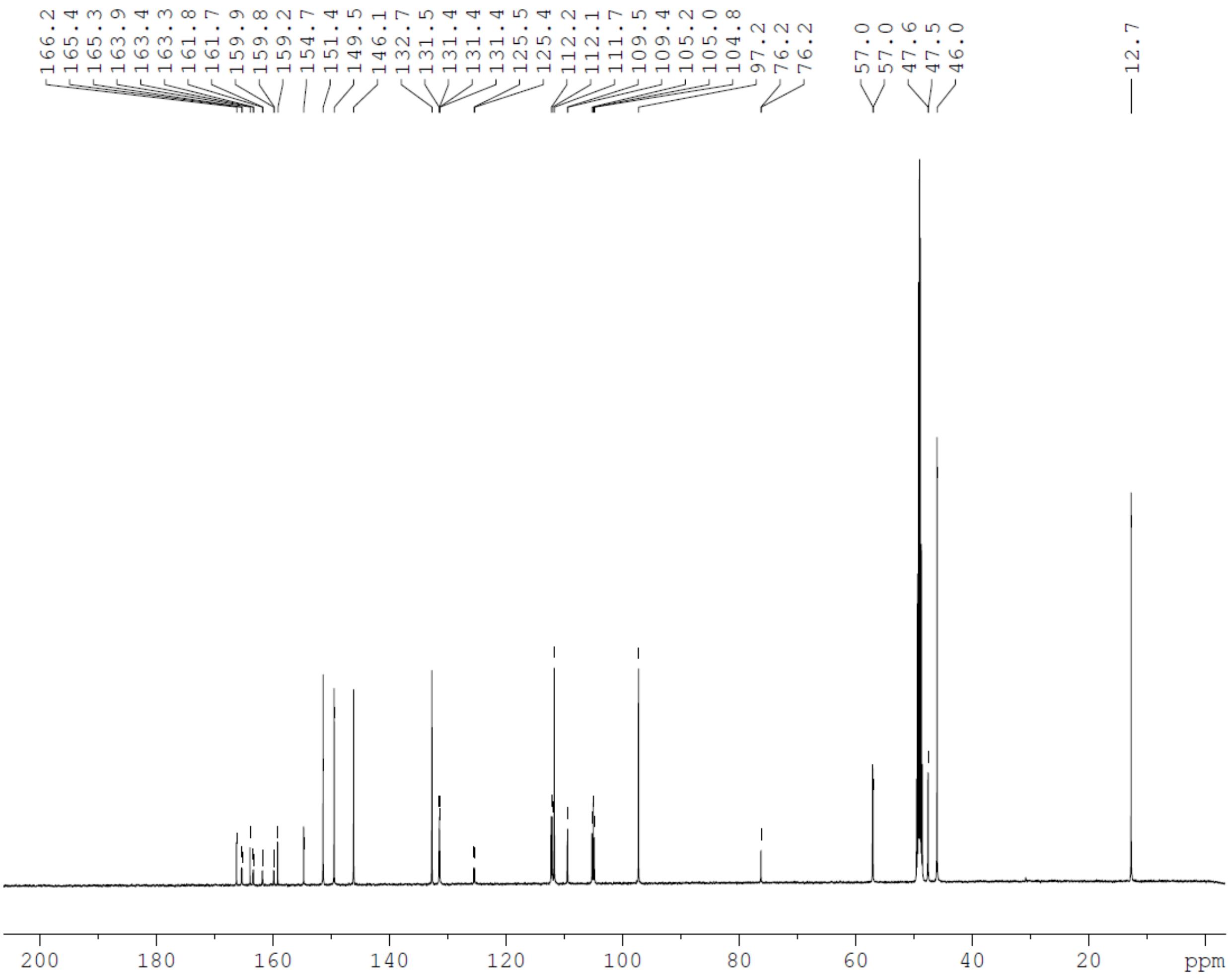

Figure S13. 125 MHz ^{13}C-NMR spectrum of compound ***1**$_R$* in CD_3OD

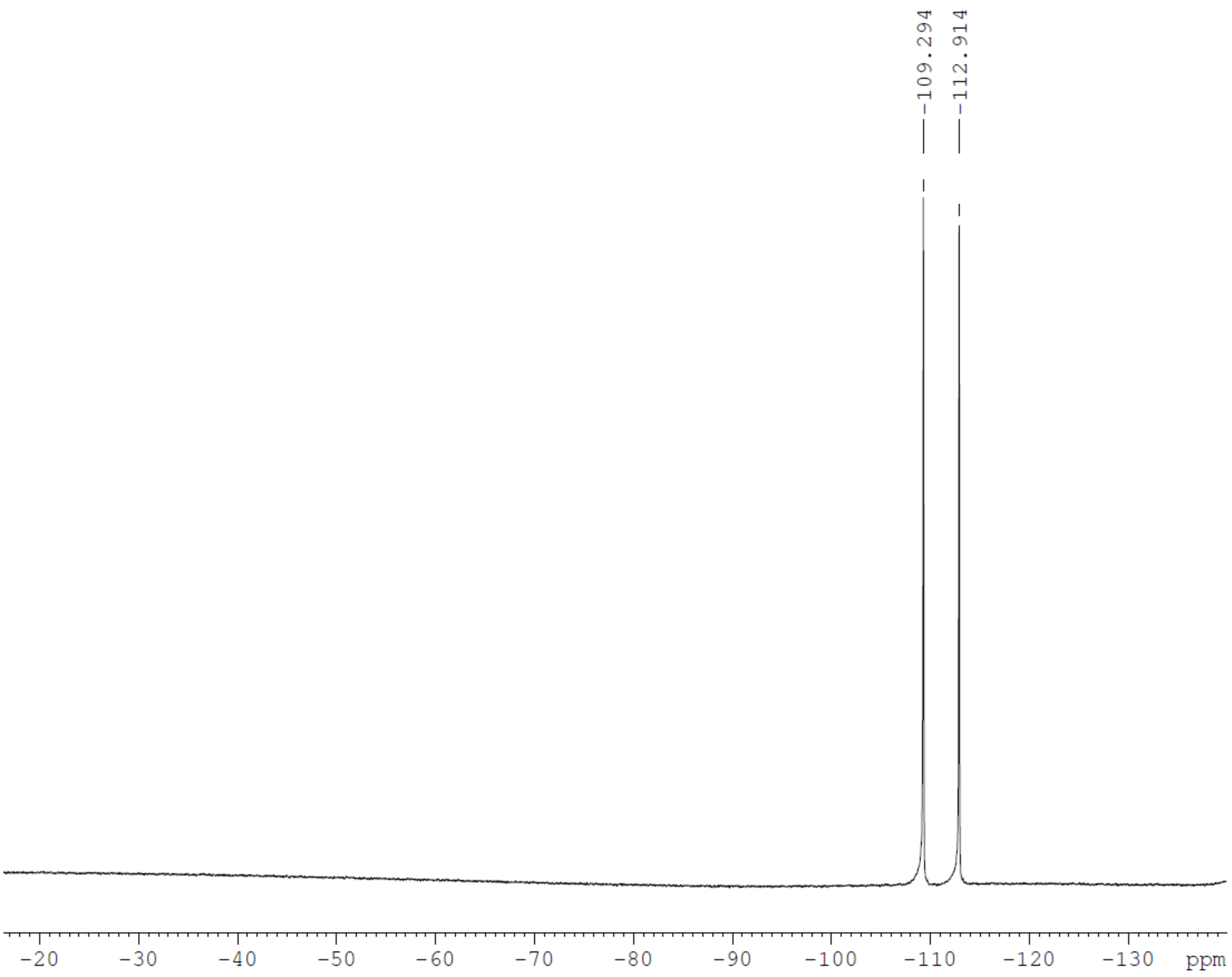

Figure S14. 376 MHz ^{19}F-NMR spectrum of compound ***1$_R$*** in CD_3OD

Analytical HPLC chromatograms

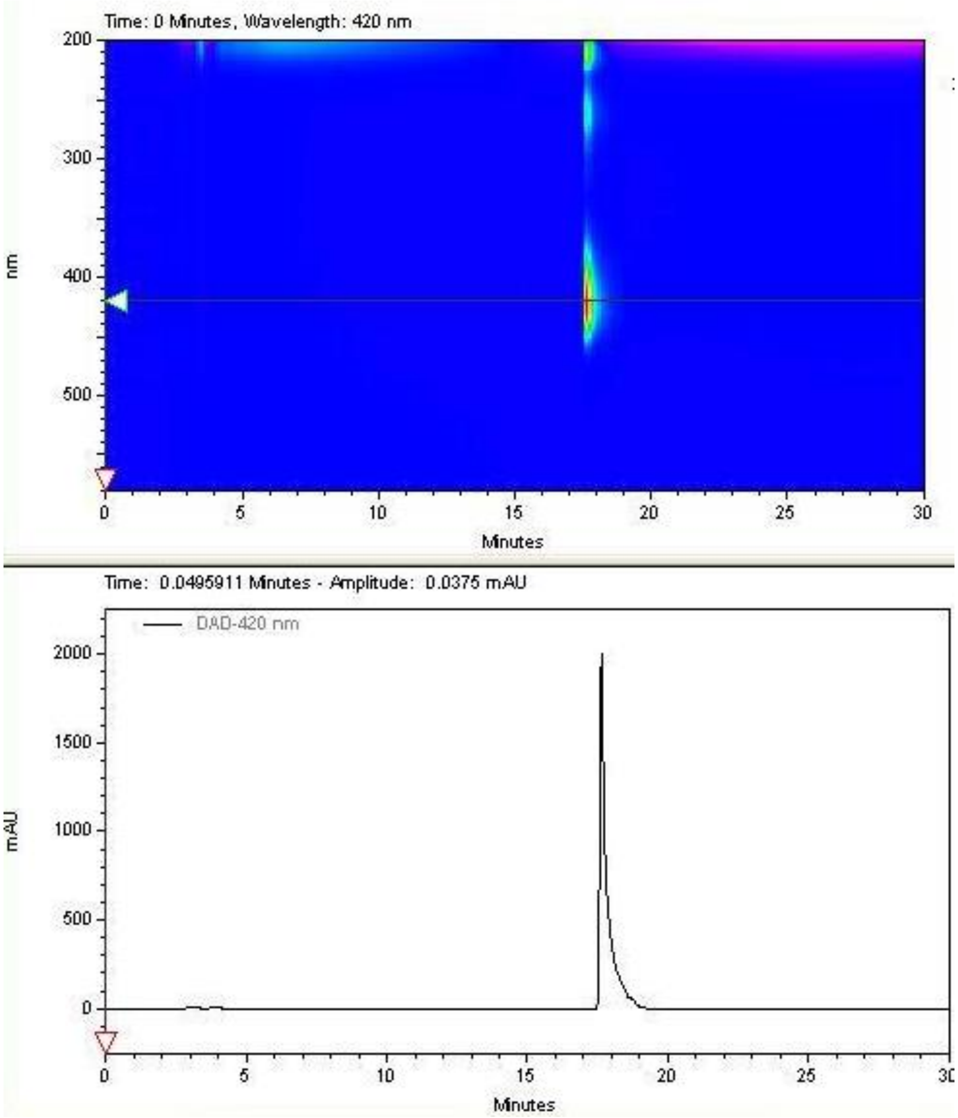

Figure S15. Analytic RP-HPLC chromatogram (diode array detector) of compound ***1*s**. HPLC conditions: mobile phase: Acetonitrile in H_2O (containing 0.1% TFA), gradient from 10% to 90%; flow rate: 1 mL/min.

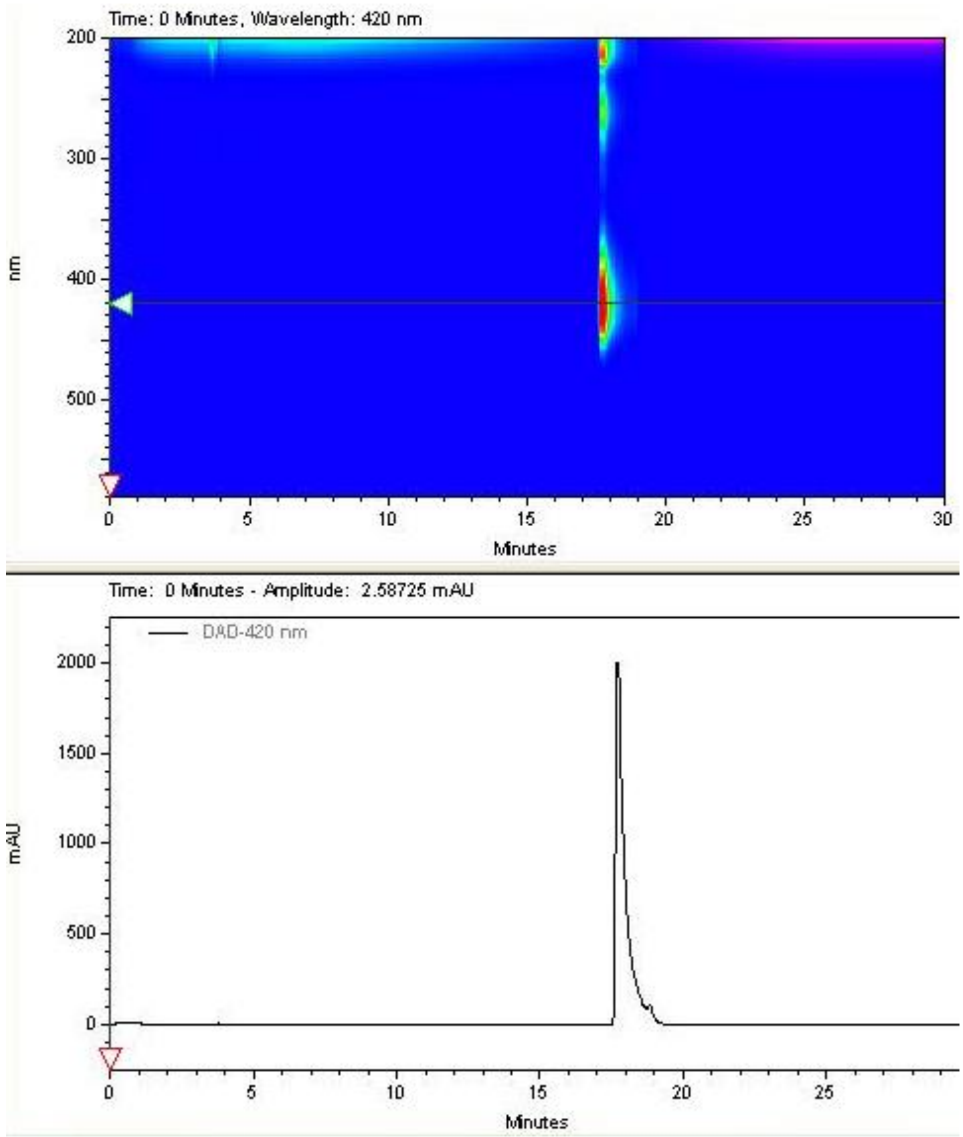

Figure S16. Analytic RP-HPLC chromatogram (diode array detector) of compound ***1**$_R$*. HPLC conditions: mobile phase: Acetonitrile in H_2O (containing 0.1% TFA), gradient from 10% to 90%; flow rate: 1 mL/min.

Absorption/Emission spectra

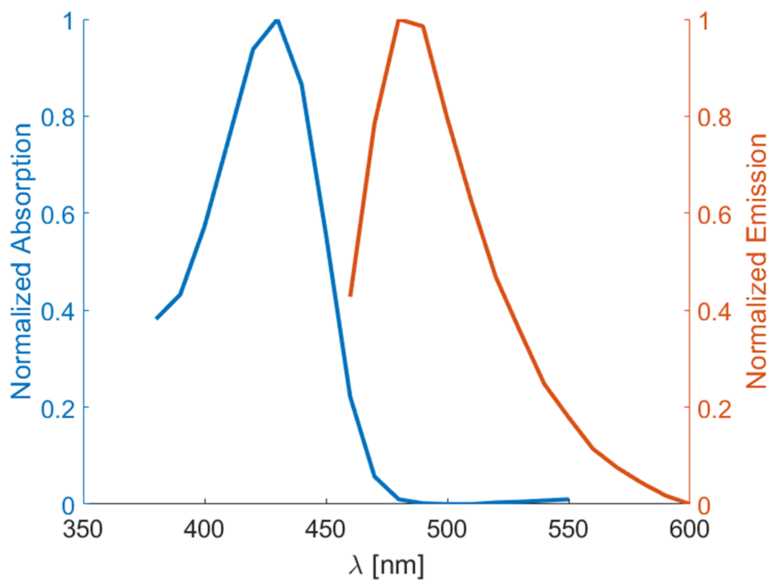

Figure S17. Normalized Absorption and emission spectra of compound ***1*****S**. The measurements were made at the concentration of 10 μM in PBS (pH 7.4). Emission spectrum was measured after excitation at 420 nm.

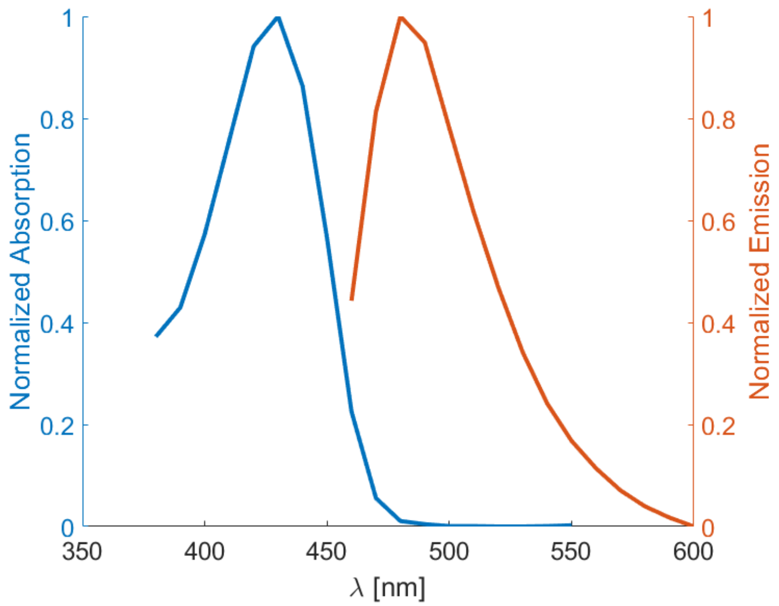

Figure S18. Normalized Absorption and emission spectra of compound ***1*****R**. The measurements were made at the concentration of 10 μM in PBS (pH 7.4). Emission spectrum was measured after excitation at 420 nm.

Yeast strains

Table S1. Strains Information: *C. albicans* strains in the panel.

Strain #	Species	Strain Name	Isogenic parental strain	Genotype	Source
**1**	*C. albicans*	T-2068	Clinical isolate	FKS1 HS1-F641A mutation	David Perlin[1]
**2**	*C. albicans*	T-2069	Clinical isolate	FKS1 HS1-S645F mutation	David Perlin[2]
**3**	*C. albicans*	T-2074	Clinical isolate	FKS1 HS1-P649H mutation	David Perlin[2]
**4**	*C. albicans*	T-2076	Clinical isolate	FKS1 HS1-S645F, HS2-R1361R/H mutation	David Perlin[2]
**5**	*C. albicans*	T-2077	Clinical isolate	FKS1 HS1-S645P mutation	David Perlin[2]
**6**	*C. albicans*	T-3528 (DPL1015)	SC5314	FKS1 HS1-S645S/P mutation	David Perlin[1]
**7**	*C. albicans*	T-3529 (DPL1016)	SC5314	FKS1 HS1-S645P mutation	David Perlin[1]
**8**	*C. albicans*	T-1 (SC5314)	WT		Judith Berman
**9**	*C. albicans*	T-736 (SN152)		leu2Δ/leu2Δ his1Δ/his1Δ arg4Δ/arg4Δ URA3/ura3Δ::imm^{434} IRO1/iro1Δ::imm^{434}	Susan Lindquist[3]
**10**	*C. albicans*	T-280 (DSY296)	Clinical isolate	TAC1-5/TAC1-5 ERG11-5/ERG11-5	Dominique Sanglard[4]
**11**	*C. albicans*	T-3463	SC5314	SCTAC1GAD1 ZnTF-GAD fusion	Judith Berman
**12**	*C. albicans*	T-783 (DSY2323)	Clinical isolate		Dominique Sanglard[5]
**13**	*C. albicans*	T-601 (VSY18)	VSY11	erg3-1::FRT/erg3-1::FRT	Dominique Sanglard[6]
**14**	*C. albicans*	T-3470	SC5314	SCUPC2GAD1 ZnTF-GAD fusion	Judith Berman
**15**	*C. albicans*	T-742 (BV11)	SN152	leu2Δ/leu2Δhis1Δ/his1Δ arg4Δ/arg4ΔURA3/ura3Δ::imm^{434} IRO1/iro1Δ::imm^{434} erg3Δ::C.d.HIS1/erg3Δ: C.m.LEU2 erg11Δ:C.d.ARG4/erg11Δ/C.d.ARG4	Susan Lindquist[3]
**16**	*C. albicans*	T-3455	SC5314	SCMRR1GAD1 ZnTF-GAD fusion	Judith Berman
**17**	*C. albicans*	T-367 (GC75)	Clinical isolate		Richard Bennett[7]
**18**	*C. albicans*	T-371 (P37037)	Clinical isolate		Richard Bennett[7]
**19**	*C. albicans*	T-363 (P87)	Clinical Isolate		Richard Bennett[8]

Table S2. Strains Information: *C. glabrata* strains in the panel.

Strain #	Species	Strain Name	Isogenic parental strain	Genotype	Source
**20**	*C. glabrata*	T-3530 (DPL1021)	ATCC90030		Judith Berman
**21**	*C. glabrata*	T-3531 (DPL1086)	ATCC90030	FKS1 HS1-S629P mutation	David Perlin[9]
**22**	*C. glabrata*	T-3536 (TGL00054)	CST109	FKS2 HS1-F659-- L664R, HS2-R1378L	Toni Gabaldon[10]
**23**	*C. glabrata*	T-3540 (TGL00107)	CST34	FKS1 HS1-S629P-P633Q	Toni Gabaldon[10]
**24**	*C. glabrata*	T-3542 (TGL00258)	CST34	FKS1 HS1-S629P	Toni Gabaldon[10]
**25**	*C. glabrata*	T-3543 (CST34)	Clinical isolate		Toni Gabaldon[10]
**26**	*C. glabrata*	T-3548 (TGL00065)	CST78	FKS1 HS1-L628I-D632N	Toni Gabaldon[10]
**27**	*C. glabrata*	T-3549 (TGL00263)	CST78	FKS2 HS1-F659S	Toni Gabaldon[10]
**28**	*C. glabrata*	T-3552 (CST78)	Clinical isolate		Toni Gabaldon[10]
**29**	*C. glabrata*	T-1545 (RBAY11-012)	Clinical isolate		Ronen Ben Ami
**30**	*C. glabrata*	T-1549 (RBAY11-016)	Clinical isolate		Ronen Ben Ami
**31**	*C. glabrata*	T-1572 (RBAY11-039)	Clinical isolate		Ronen Ben Ami
**32**	*C. glabrata*	T-1575 (RBAY11-042)	Clinical isolate		Ronen Ben Ami
**33**	*C. glabrata*	T-1582 (RBAY11-0482)	Clinical isolate		Ronen Ben Ami
**34**	*C. glabrata*	T-1597 (RBAY11-064)	Clinical isolate		Ronen Ben Ami
**35**	*C. glabrata*	T-1603 (RBAY11-070)	Clinical isolate		Ronen Ben Ami
**36**	*C. glabrata*	T-1610 (RBAY11-076)	Clinical isolate		Ronen Ben Ami
**37**	*C. glabrata*	T-1044 (RBAY11-295)	Clinical isolate		Ronen Ben Ami
**38**	*C. glabrata*	T-1045 (RBAY11-191)	Clinical isolate		Ronen Ben Ami
**39**	*C. glabrata*	T-1046 (1708)	Clinical isolate		Ronen Ben Ami
**40**	*C. glabrata*	T-1338	Clinical isolate		Judith Berman
**41**	*C. glabrata*	T-1339	Clinical isolate		Judith Berman
**42**	*C. glabrata*	T-1340	Clinical isolate		Judith Berman
**43**	*C. glabrata*	T-1341	Clinical isolate		Judith Berman

Table S3. Strains Information: *C. parapsilosis* strains in the panel.

Strain #	Species	Strain Name	Isogenic parental strain	Source
**44**	*C. parapsilosis*	T-1537 (RBAY11004)	Clinical isolate	Ronen Ben Ami
**45**	*C. parapsilosis*	T-1539 (RBAY11006)	Clinical isolate	Ronen Ben Ami
**46**	*C. parapsilosis*	T-1541 (RBAY11008)	Clinical isolate	Ronen Ben Ami
**47**	*C. parapsilosis*	T-1554 (RBAY11021)	Clinical isolate	Ronen Ben Ami
**48**	*C. parapsilosis*	T-1556 (RBAY11023)	Clinical isolate	Ronen Ben Ami
**49**	*C. parapsilosis*	T-1557 (RBAY11024)	Clinical isolate	Ronen Ben Ami
**50**	*C. parapsilosis*	T-1561 (RBAY11028)	Clinical isolate	Ronen Ben Ami
**51**	*C. parapsilosis*	T-1565 (RBAY11032)	Clinical isolate	Ronen Ben Ami
**52**	*C. parapsilosis*	T-1576 (RBAY11043)	Clinical isolate	Ronen Ben Ami
**53**	*C. parapsilosis*	T-1592 (RBAY11059)	Clinical isolate	Ronen Ben Ami
**54**	*C. parapsilosis*	T-1601 (RBAY11068)	Clinical isolate	Ronen Ben Ami
**55**	*C. parapsilosis*	T-1602 (RBAY11069)	Clinical isolate	Ronen Ben Ami
**56**	*C. parapsilosis*	T-1618 (RBAY11084)	Clinical isolate	Ronen Ben Ami
**57**	*C. parapsilosis*	T-1619 (RBAY11085)	Clinical isolate	Ronen Ben Ami
**58**	*C. parapsilosis*	T-54 (YA053)	Clinical isolate	Catherine Bendel[11]
**59**	*C. parapsilosis*	T-55 (A041)	Clinical isolate	Catherine Bendel[11]
**60**	*C. parapsilosis*	T-56 (4961)	Clinical isolate	Catherine Bendel[11]

Minimal Inhibitory Concentration (MIC) tables.

MIC values in tables S4-S6 were determined using the broth double-dilution method between the concentrations 64 µg/mL and 1/64 (0.015625) µg/mL. Cells were grown in YPAD medium at 30°C for 24 h. Each concentration was tested in triplicate, and results were confirmed by two independent sets of experiments. MIC values were defined as the point at which there is no visible growth compared to the no-compound wells.

Table S4. Minimal Inhibitory Concentration (MIC) values of probes ***1*** $_S$, ***1*** $_R$ and fluconazole for the *C. albicans* strains in the panel.

Strain #	Compound MIC [µg/mL]		
	1 $_S$	***1*** $_R$	**Fluconazole**
**1**	0.03125	2	0.25
**2**	0.03125	2	0.25
**3**	>64	>64	>64
**4**	32	>64	>64
**5**	8	>64	64
**6**	0.03125	2	0.25
**7**	0.015625	2	0.25
**8**	0.03125	4	1
**9**	0.03125	4	0.5
**10**	>64	>64	>64
**11**	1	>64	8
**12**	8	>64	>64
**13**	>64	>64	>64
**14**	0.03125	8	1
**15**	>64	>64	>64
**16**	0.125	8	4
**17**	0.0625	4	1
**18**	0.015625	4	0.5
**19**	0.125	2	0.25

Table S5. Minimal Inhibitory Concentration (MIC) values of probes ***1**$_S$*, ***1**$_R$* and fluconazole for the *C. glabrata* strains in the panel.

Strain #	Compound MIC [µg/mL]		
	***1**$_S$*	***1**$_R$*	**Fluconazole**
**20**	8	>64	16
**21**	8	>64	16
**22**	8	>64	16
**23**	8	>64	>64
**24**	32	>64	>64
**25**	8	>64	16
**26**	2	>64	32
**27**	32	>64	>64
**28**	8	>64	16
**29**	1	>64	16
**30**	4	>64	16
**31**	4	>64	16
**32**	4	>64	16
**33**	4	>64	16
**34**	8	>64	16
**35**	2	>64	16
**36**	4	>64	16
**37**	2	>64	16
**38**	>64	>64	>64
**39**	>64	>64	>64
**40**	16	>64	>64
**41**	>64	>64	>64
**42**	16	>64	>64
**43**	>64	>64	>64

Table S6. Minimal Inhibitory Concentration (MIC) values of probes ***1**$_S$*, ***1**$_R$* and fluconazole for the *C. parapsilosis* strains in the panel.

Strain #	Compound MIC [µg/mL]		
	***1**$_S$*	***1**$_R$*	**Fluconazole**
**44**	2	>64	8
**45**	4	>64	32
**46**	0.25	8	1
**47**	1	>64	8
**48**	0.0625	8	1
**49**	0.25	>64	4
**50**	0.25	32	4
**51**	0.0625	16	1
**52**	0.0625	4	1
**53**	0.03125	2	1
**54**	0.25	16	2
**55**	0.5	>64	4
**56**	0.125	8	1
**57**	0.0625	2	1
**58**	0.25	>64	4
**59**	<0.015625	2	1
**60**	0.25	>64	4

Table S7. Information about the eight strains used to validate the developed method for species identification.

Strain #	Species	Strain Name	Isogenic parental strain	Source
**V1**	*C. albicans*	ATCC24433	Clinical isolate	ATCC
**V2**	*C. albicans*	ATCC90028	Clinical isolate	ATCC
**V3**	*C. albicans*	SN95	Clinical isolate	Judith Berman
**V4**	*C. glabrata*	ATCC2001	Clinical isolate	ATCC
**V5**	*C. glabrata*	ATCC66032	Clinical isolate	ATCC
**V6**	*C. glabrata*	BG2 (T-50)	Clinical isolate	Judith Berman
**V7**	*C. parapsilosis*	ATCC22019	Clinical isolate	ATCC
**V8**	*C. parapsilosis*	ATCC90018	Clinical isolate	ATCC

Table S8. Minimal Inhibitory Concentration (MIC) values of probes $\mathbf{1}_S$, $\mathbf{1}_R$ and fluconazole determined for the eight strains used to validate the developed method for species identification.

Strain #	Compound MIC [µg/mL]		
	$\mathbf{1}_S$	$\mathbf{1}_R$	**Fluconazole**
**V1**	0.0625	>64	1
**V2**	0.03125	4	0.5
**V3**	0.015625	16	0.5
**V4**	2	>64	8
**V5**	2	>64	16
**V6**	8	>64	16
**V7**	1	>64	16
**V8**	0.03125	8	1

MIC values were determined using the broth double-dilution method between the concentrations 64 µg/mL and 1/64 (0.015625) µg/mL. Cells were grown in YPAD medium at 30°C for 24 h. Each concentration was tested in triplicate, and results were confirmed by two independent sets of experiments. MIC values were defined as the point at which there is no visible growth compared to the no-compound wells.

References

1. Ben-Ami, R., Garcia-Effron, G., Lewis, R.E., Gamarra, S., Leventakos, K., Perlin, D.S., and Kontoyiannis, D.P. (2011). Fitness and Virulence Costs of Candida albicans FKS1 Hot Spot Mutations Associated With Echinocandin Resistance. J. Infect. Dis. *204*, 626–635. https://doi.org/10.1093/INFDIS/JIR351.
2. Garcia-Effron, G., Park, S., and Perlin, D.S. (2009). Correlating echinocandin MIC and kinetic inhibition of fks1 mutant glucan synthases for Candida albicans: Implications for interpretive breakpoints. Antimicrob. Agents Chemother. *53*, 112–122. https://doi.org/10.1128/AAC.01162-08.
3. Vincent, B.M., Lancaster, A.K., Scherz-Shouval, R., Whitesell, L., and Lindquist, S. (2013). Fitness Trade-offs Restrict the Evolution of Resistance to Amphotericin B. PLOS Biol. *11*, e1001692. https://doi.org/10.1371/JOURNAL.PBIO.1001692.
4. Maccallum, D.M., Coste, A., Ischer, F., Jacobsen, M.D., Odds, F.C., and Sanglard, D. (2010). Genetic Dissection of Azole Resistance Mechanisms in Candida albicans and Their Validation in a Mouse Model of Disseminated Infection. Antimicrob. Agents Chemother. *54*, 1476–1483. https://doi.org/10.1128/AAC.01645-09.
5. Coste, A., Selmecki, A., Forche, A., Diogo, D., Bougnoux, M.E., D'Enfert, C., Berman, J., and Sanglard, D. (2007). Genotypic Evolution of Azole Resistance Mechanisms in Sequential Candida albicans Isolates. Eukaryot. Cell *6*, 1889. https://doi.org/10.1128/EC.00151-07.
6. Vale-Silva, L.A., Coste, A.T., Ischer, F., Parker, J.E., Kelly, S.L., Pinto, E., and Sanglard, D. (2012). Azole Resistance by Loss of Function of the Sterol Δ5,6-Desaturase Gene (ERG3) in Candida albicans Does Not Necessarily Decrease Virulence. Antimicrob. Agents Chemother. *56*, 1960. https://doi.org/10.1128/AAC.05720-11.
7. Wu, W., Lockhart, S.R., Pujol, C., Srikantha, T., and Soll, D.R. (2007). Heterozygosity of genes on the sex chromosome regulates Candida albicans virulence. Mol. Microbiol. *64*, 1587–1604. https://doi.org/10.1111/J.1365-2958.2007.05759.X.
8. Blignaut, E., Pujol, C., Lockhart, S., Joly, S., and Soll, D.R. (2002). Ca3 Fingerprinting of Candida albicans Isolates from Human Immunodeficiency Virus-Positive and Healthy Individuals Reveals a New Clade in South Africa. J. Clin. Microbiol. *40*, 826. https://doi.org/10.1128/JCM.40.3.826-836.2002.
9. Garcia-Effron, G., Lee, S., Park, S., Cleary, J.D., and Perlin, D.S. (2009). Effect of Candida glabrata FKS1 and FKS2 mutations on echinocandin sensitivity and kinetics of 1,3-β-D-glucan synthase: Implication for the existing susceptibility breakpoint. Antimicrob. Agents Chemother. *53*, 3690–3699. https://doi.org/10.1128/AAC.00443-09.
10. Carreté, L., Ksiezopolska, E., Pegueroles, C., Gómez-Molero, E., Saus, E., Iraola-Guzmán, S., Loska, D., Bader, O., Fairhead, C., and Gabaldón, T. (2018). Patterns of Genomic Variation in the Opportunistic Pathogen Candida glabrata Suggest the Existence of Mating and a Secondary Association with Humans. Curr. Biol. *28*, 15-27.e7. https://doi.org/10.1016/j.cub.2017.11.027.
11. Reiss, E., Lasker, B.A., Lott, T.J., Bendel, C.M., Kaufman, D.A., Hazen, K.C.,

Wade, K.C., Mcgowan, K.L., and Lockhart, S.R. (2012). Genotyping of Candida parapsilosis from three neonatal intensive care units (NICUs) using a panel of five multilocus microsatellite markers: Broad genetic diversity and a cluster of related strains in one NICU. Infect. Genet. Evol. *12*, 1654–1660. https://doi.org/10.1016/j.meegid.2012.06.012.
